# Mathematical modeling and optimal control of immune responses in multiple sclerosis

**DOI:** 10.1371/journal.pone.0349131

**Published:** 2026-11-10

**Authors:** Mo’tassem Al-arydah

**Affiliations:** Department of Mathematics, Khalifa University, Abu Dhabi, United Arab Emirates; Gulf University for Science and Technology, KUWAIT

## Abstract

Multiple sclerosis (MS) is a chronic autoimmune disease characterized by immune-mediated destruction of the myelin sheath in the central nervous system. In this work, we develop and analyze a mathematical model describing the interactions among myelin, autoreactive immune cells, regulatory immune cells, and treatment. A fast-time reduction of the slow–fast model yields an analytically tractable immune subsystem that captures the principal immune interactions. We establish the existence and stability properties of the immune-free and interior equilibria and derive the autoimmune activation number $R_{0}$ governing immune persistence. The analysis reveals a treatment-dependent threshold structure separating two distinct dynamical regimes. For $R_{0}>1$, the reduced immune subsystem admits an interior equilibrium that is a nonlinear center surrounded by closed periodic orbits, whereas for $R_{0}<1$, the immune-free equilibrium is globally asymptotically stable. Numerical simulations of the full model exhibit recurrent inflammatory oscillations under insufficient treatment and suppression of autoimmune activity under sufficiently strong treatment. Analytical sensitivity indices and global sensitivity analysis using LHS–PRCC identify immune activation and autoreactive immune-cell removal as the dominant mechanisms influencing the autoimmune activation threshold. An optimal control framework is developed to balance autoimmune suppression, cumulative treatment intensity, and treatment adjustment costs. Numerical results indicate that the computed treatment strategies can reduce autoimmune burden while accounting for treatment-related costs. Overall, the proposed framework provides insights into the interplay between immune regulation, myelin damage, and treatment, highlighting the role of treatment-dependent thresholds and optimized interventions in controlling autoimmune activity in multiple sclerosis.

## 1 Introduction

MS is a chronic autoimmune disorder characterized by immune-mediated destruction of the myelin sheath in the central nervous system (CNS). This demyelination process, together with associated axonal damage, disrupts neural communication and leads to a wide range of neurological impairments. Despite extensive clinical and experimental research, the underlying mechanisms driving disease progression, relapse patterns, and long-term neurological decline remain incompletely understood, particularly in relation to the complex interplay between immune activation and regulatory processes [1].

Mathematical modeling has become an essential tool in biomedical research, providing a systematic framework for analyzing complex biological systems, testing hypotheses, and informing therapeutic strategies. Such approaches have been successfully applied to diseases including cancer, HIV, and infectious diseases, where they have contributed to identifying key regulatory mechanisms and optimizing treatment protocols [2,3]. In contrast, multiple sclerosis has received comparatively limited attention from a mathematical modeling perspective, despite its inherently immune-driven nature and clinical complexity. This underrepresentation highlights a significant opportunity for the development of quantitative frameworks capable of advancing both theoretical understanding and treatment design.

Existing mathematical models of MS dynamics have primarily focused on specific aspects of the disease, such as T-cell activation, lesion formation, or simplified immune–myelin interactions [4,5]. While these studies have provided valuable insights into individual mechanisms, they generally do not simultaneously incorporate damage-dependent immune activation, regulatory immune responses, treatment-induced modulation, and optimal control within a single analytically tractable framework. The present model addresses these aspects simultaneously, allowing the investigation of treatment-dependent threshold dynamics and long-term immune behavior.

Recent advances in immunology emphasize that MS cannot be fully understood through a purely T-cell–centric perspective, as regulatory immune mechanisms and broader immune interactions play a crucial role in disease progression [6,7]. At the same time, clinical evidence demonstrates that early and sustained treatment can significantly alter disease trajectories. However, there remains a lack of mathematical models capable of quantitatively linking immune dynamics, therapeutic intervention, and long-term neurological outcomes. Addressing this gap is essential for improving both mechanistic understanding and treatment optimization.

In this work, we develop and analyze a novel mathematical model that integrates damage-driven immune activation, regulatory immune response, and treatment-induced modulation within a unified framework. The model explicitly captures the coupling between myelin degradation and immune activation, leading to a nonlinear feedback structure that governs disease progression. A quasi–steady-state reduction yields a reduced system that remains analytically tractable while preserving the essential biological mechanisms.

The key contribution of this work is the characterization of a treatment-dependent threshold structure that links treatment intensity, immune activation, and long-term disease dynamics. Specifically, we show that the model exhibits two distinct dynamical regimes separated by a critical treatment level: one characterized by persistent oscillatory autoimmune activity and the other by immune suppression and convergence to the immune-free state. Thus, within the proposed modeling framework, treatment governs the qualitative behavior of the system.

More broadly, the purpose of this work is to provide a mechanistic framework for understanding how myelin damage, immune activation, immune regulation, and treatment interact to shape disease progression in multiple sclerosis. Rather than evaluating a specific clinical therapy, the treatment component is incorporated as a generic intervention mechanism that allows us to investigate treatment thresholds, long-term disease dynamics, and the potential impact of treatment on autoimmune activity and myelin preservation.

We further establish fundamental mathematical properties of the model, including existence, uniqueness, nonnegativity, and boundedness of solutions. The reduced system is analyzed to determine the existence and stability of the disease-free, boundary, and interior equilibria. In addition, an optimal control problem is formulated to investigate how time-dependent treatment strategies influence disease burden while accounting for treatment costs. The resulting optimality system is solved numerically to illustrate the effectiveness of treatment in suppressing inflammatory dynamics and preserving neuronal function.

The remainder of the paper is organized as follows. We first formulate the mathematical model and establish its well-posedness. A reduced system is then derived and analyzed to characterize equilibrium behavior and threshold dynamics. Next, an optimal control framework is developed and analyzed. Numerical simulations are presented to illustrate the theoretical results and explore the impact of key parameters. Finally, we conclude with a summary of the main findings and directions for future research.

## 2 Model formulation and assumptions

We begin by making the following biological and modeling assumptions:

a. The model represents an internal autoimmune process with no external infection, environmental transmission, or migration.
b. Autoreactive immune cells become activated in response to myelin damage in the central nervous system.
c. Regulatory immune cells suppress the proliferation and inflammatory activity of autoreactive immune cells.
d. Myelin is subject to intrinsic turnover and immune-mediated degradation.
e. Remyelination and demyelination occur on a slower physiological timescale than immune cell activation and suppression.
f. Homogeneous mixing is assumed, meaning that cellular interactions occur uniformly within the system. The homogeneous mixing assumption is a standard approximation in compartmental models and implies that interactions among immune cells and myelin are represented by average population densities rather than explicit spatial distributions. Although multiple sclerosis exhibits spatial heterogeneity and localized lesions, the present study focuses on the overall immune-myelin dynamics and treatment effects at the population level.
g. Myelin has a theoretical upper bound $M_{\max}$ corresponding to a fully healthy state, which is not exceeded in biological conditions.

The system is divided into three interacting compartments: healthy myelin in the nervous system *M*(*t*), autoreactive immune cells (e.g., pathogenic T-cells) *I*(*t*), and regulatory immune cells (e.g. T_reg_ cells) *R*(*t*).

Autoreactive immune activation is assumed to increase with the level of myelin damage. We define the damage variable by


$$\begin{array}{c} D=M_{\max}-M, \end{array}$$

where $M_{\max}$ denotes the maximum healthy myelin level and *M* denotes the current healthy myelin level. Thus, *D* = 0 corresponds to the absence of myelin damage, whereas larger values of *D* represent increasing degrees of demyelination. This choice is biologically motivated by the observation that demyelination increases the exposure of myelin antigens to the immune system, thereby enhancing autoreactive immune responses. Therefore, it is natural to assume that the immune activation rate is an increasing function of the damage level *D*.

The immune activation rate is modeled by the saturating function


$$\begin{array}{c} \eta (D)=\eta_{\max}\frac{D}{\omega +D},\;\eta_{\max}>0,\;\omega >0, \end{array}$$

where $\eta_{\max}$ is the maximum activation rate and ω is the half-saturation constant. This formulation reflects the biological observation that greater myelin damage exposes more self-antigens, thereby promoting autoreactive immune activation, while the activation rate approaches a finite maximum for large damage levels.

The saturating form of η(D) reflects the biological observation that increasing myelin damage promotes autoreactive immune activation through the release of damage-associated signals, antigen presentation, and inflammatory mediators. However, immune activation cannot increase indefinitely because of finite immune-cell availability, receptor saturation, signaling constraints, and regulatory feedback. Although no direct experimental evidence establishes this exact functional relationship in multiple sclerosis, a Michaelis–Menten/Holling type II response provides a biologically plausible phenomenological approximation while ensuring a bounded activation rate.

Healthy myelin *M*(*t*) is naturally regenerated at rate α, and is degraded (i) intrinsically at rate δM and (ii) through immune-mediated attack at rate βIM. Autoreactive immune cells *I*(*t*) proliferate at rate η(D)I, are suppressed by regulatory cells at rate μRI, and are removed by natural death and treatment a*t* rate (θ+τ)I, where θ denotes natural cell death and τ reflects therapeutic intervention. Regulatory immune cells *R*(*t*) expand proportionally to autoreactive cell presence at rate ρIR and decay naturally at rate λR. The functional capacity of neurons *N*(*t*) deteriorates when myelin falls below its maximal healthy level $M_{\max}$. The degradation of neurons is proportional to the damage level *D* and the current functional capacity *N*(*t*).

In the present formulation, *N*(*t*) is treated as a downstream measure of cumulative neurological damage. Neuronal repair or recovery, as well as feedback from neuronal damage to the immune dynamics, are not included and are left for future extensions of the model.

Consistent with the biological assumptions stated above, the model is intended to provide a mechanistic description of the interactions among myelin damage, autoreactive immune activation, regulatory immune responses, and treatment intervention in multiple sclerosis. The model structure is motivated by the current biological understanding of disease progression, whereby demyelination promotes immune activation, regulatory immune cells suppress excessive inflammation, and treatment-induced suppression reduces autoreactive immune activity. Although the model does not include all immunological pathways involved in multiple sclerosis, it incorporates the principal mechanisms believed to drive disease progression while remaining analytically tractable.

A complete list of variables and parameters is summarized in Table 1, and the corresponding flowchart of the model is presented in Fig 1.

**Table 1:** Description of model parameters.

Parameter	Description
α	Natural regeneration rate of healthy myelin
β	Rate of immune-mediated myelin degradation
δ	Natural degradation rate of myelin
$\eta_{\max}$	Maximum activation rate of autoreactive immune cells
ω	Half-saturation constant in the activation function η(D)
μ	Suppression rate of autoreactive immune cells by regulatory cells
θ	Natural death rate of autoreactive immune cells
τ	Treatment-induced removal rate of autoreactive immune cells
ρ	Proliferation rate of regulatory immune cells in the presence of inflammation
λ	Natural decay rate of regulatory immune cells
$M_{\max}$	Maximum attainable level of healthy myelin
ε	Small parameter representing the slower myelin dynamics relative to immune responses
γ	Rate of neuronal loss due to myelin deficiency

**Fig 1:**
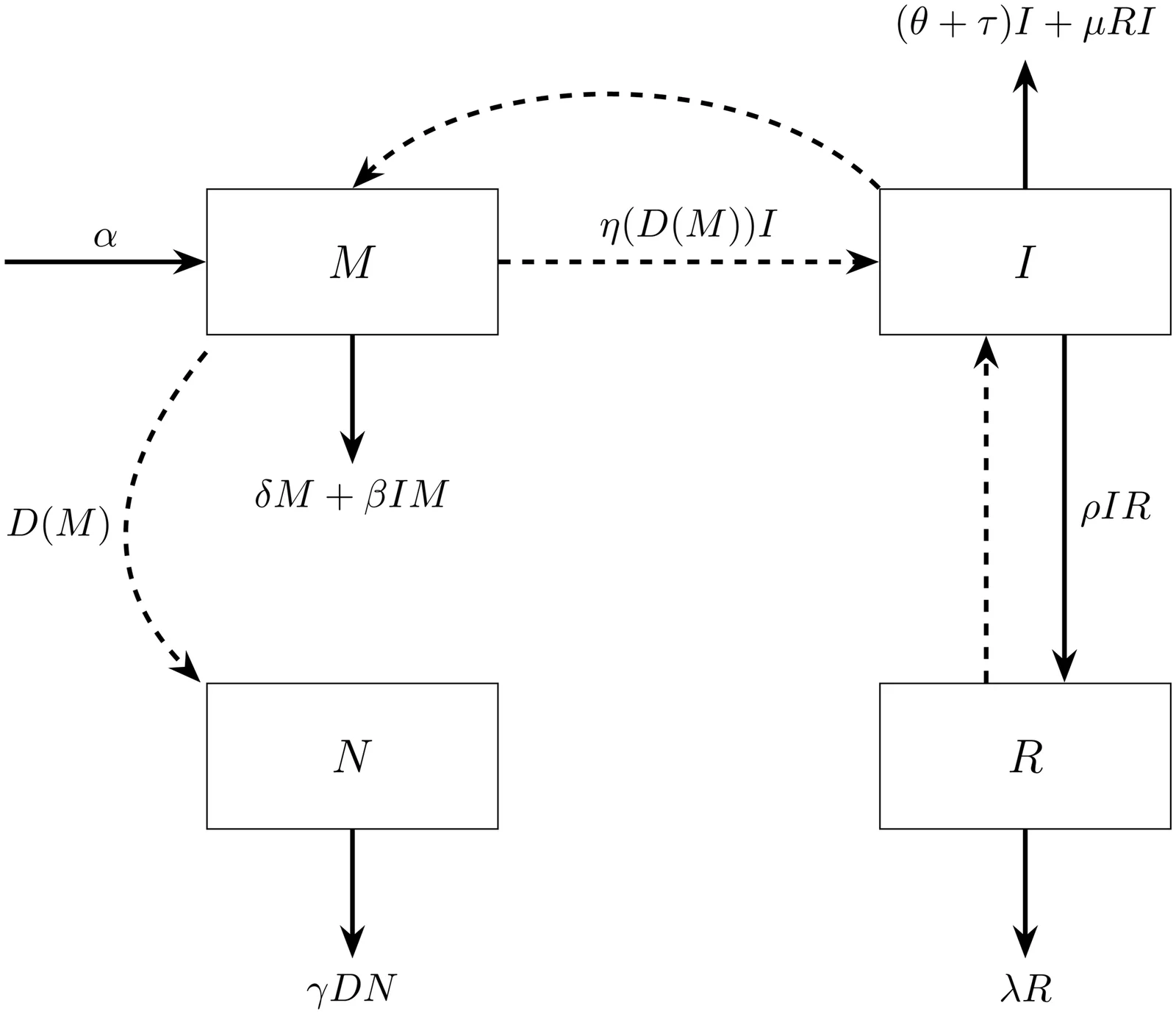
Flowchart of the proposed multiple sclerosis model. The damage variable is defined in the text by $D=M_{\max}-M$. Healthy myelin *M* is regenerated at rate α, degraded naturally at rate δM, and damaged by autoreactive immune cells at rate βIM. Myelin damage activates autoreactive immune cells through the damage-dependent term η(D)I. Regulatory immune cells suppress autoreactive immune cells at rate μRI, expand at rate ρIR, and decay at rate λR. Neuronal capacity *N* declines due to myelin damage at rate γDN.

The model resulting from these assumptions is given by the following system of ordinary differential equations:


$$\begin{array}{cc} \frac{dM}{dt} & =\varepsilon (\alpha -\beta IM-\delta M), \end{array}$$ (1)

$$\begin{array}{cc} \frac{dI}{dt} & =(\eta (D)-\mu R-(\theta +\tau ))I, \end{array}$$ (2)

$$\begin{array}{cc} \frac{dR}{dt} & =(\rho I-\lambda )R, \end{array}$$ (3)

$$\begin{array}{cc} \frac{dN}{dt} & =-\varepsilon \gamma DN \end{array}$$ (4)

where 0<ε≪1 represents the slower adaptation of myelin and neurons compared to the fast immune (*I*) and regulatory (*R*) cell populations.

The small parameter ε reflects the separation of biological time scales between the relatively fast immune dynamics and the slower processes of myelin turnover and neuronal decline. This time-scale separation motivates the fast-time reduction used later in the analysis and allows the reduced system to retain the principal biological mechanisms while remaining analytically tractable.

### 2.1 Ethics Statement

This study is based exclusively on mathematical modeling and numerical simulations. No experiments involving human participants or animals were conducted by the author.

### 2.2 Use of generative artificial intelligence

ChatGPT (OpenAI) was used to assist with language editing and improving the clarity of the manuscript. The author reviewed and edited the AI-assisted text and takes full responsibility for the accuracy and integrity of the published content. Fig 2 was generated with the assistance of ChatGPT.

**Fig 2:**
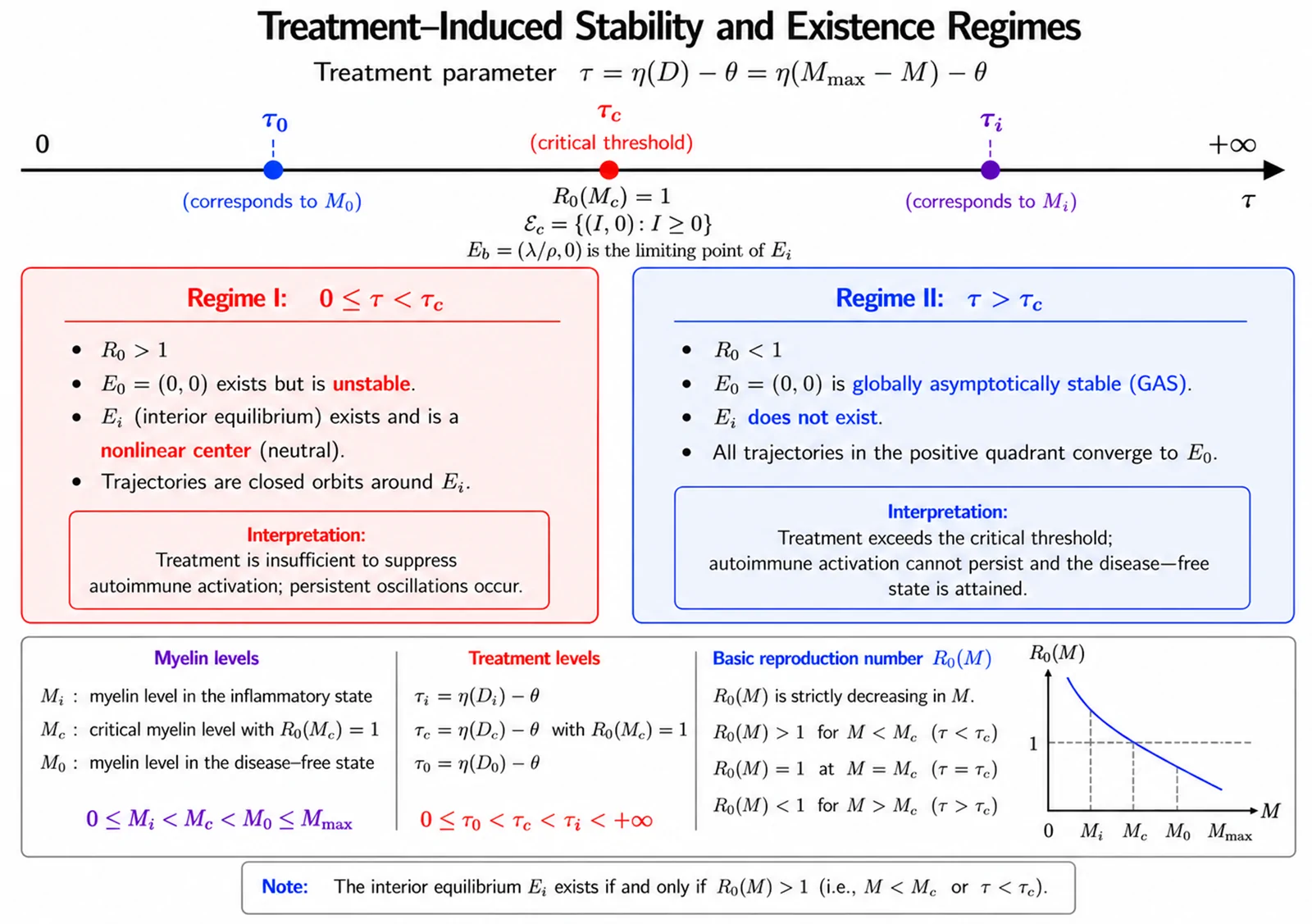
Schematic illustration of the treatment-dependent stability and threshold structure of the reduced immune subsystem. The critical treatment threshold $\tau_{c}$ separates two qualitatively distinct dynamical regimes. For $\tau <\tau_{c}$ (equivalently, ${ℛ}_{0}>1$), the interior equilibrium $E_{i}$ exists as a nonlinear center and is surrounded by closed periodic orbits, representing sustained autoimmune oscillations. At the critical threshold $\tau =\tau_{c}$ (equivalently, ${ℛ}_{0}=1$), the system possesses the continuum of boundary equilibria ${ℰ}_{c}=\{(I,0):I\geq 0\}$, and $E_{b}=(\lambda /\rho ,0)$ is the limiting position of $E_{i}$ as $\tau \to \tau_{c}^{-}$. For $\tau >\tau_{c}$ (equivalently, ${ℛ}_{0}<1$), the interior equilibrium no longer exists, and the immune-free equilibrium *E*_0_ is globally asymptotically stable, with all trajectories converging to *E*_0_.

### 2.3 Existence and uniqueness of solution

For the system of ordinary differential equations (1)–(4), we denote the right-hand sides by the vector field


$$\begin{array}{c} 𝐅(t,𝐗),\;𝐗(t)=(M(t),\;I(t),\;R(t),\;N(t){)}^{⊤}. \end{array}$$

To ensure well-posedness, we assume that **F** is continuous in *t* and locally Lipschitz with respect to **X** on ${\mathbb{R}}_{+}^{4}$. The presence of the small parameter 0<ε≪1 does not affect these regularity properties. Indeed, all polynomial and bilinear terms (such as *IM*, *RI*, and *DN*) are smooth and therefore locally Lipschitz on ${\mathbb{R}}_{+}^{4}$.

Moreover, since $D=M_{\max}-M$ is a linear function of *M*, it is smooth. The function η(D) is continuously differentiable and bounded for D≥0 (in particular, $0\leq \eta (D)\leq \eta_{\max}$), and hence is locally Lipschitz. Consequently, **F**(*t*,**X**) is locally Lipschitz with respect to **X** on any biologically relevant domain where $0\leq M\leq M_{\max}$.

Therefore, by the Picard–Lindelöf (Cauchy–Lipschitz) theorem, for any initial condition


$$\begin{array}{c} 𝐗(0)\in {\mathbb{R}}_{+}^{4}, \end{array}$$

there exists a unique local solution


$$\begin{array}{c} 𝐗(t)=(M(t),\;I(t),\;R(t),\;N(t){)}^{⊤} \end{array}$$

defined on some interval [0,*T*_1_), where *T*_1_ > 0 is the maximal time of existence.

### 2.4 Nonnegativity of Solutions

**Theorem 1.** *Assume the initial data are nonnegative,*


$$\begin{array}{c} M(0)\geq 0,\;I(0)\geq 0,\;R(0)\geq 0,\;N(0)\geq 0. \end{array}$$

*Then the corresponding solution satisfies*


$$\begin{array}{c} M(t)\geq 0,\;I(t)\geq 0,\;R(t)\geq 0,\;N(t)\geq 0\;\text{for all }t\in [0,T). \end{array}$$

*Proof.* We show that the nonnegative orthant is forward invariant.

On the boundary *M* = 0,


$$\begin{array}{c} \dot{M}{|}_{M=0}=\varepsilon \alpha >0. \end{array}$$

Thus, *M*(*t*) cannot cross into negative values.

The *I*-equation has the form


$$\begin{array}{c} \dot{I}=(\eta (D)-\mu R-(\theta +\tau ))I, \end{array}$$

so


$$\begin{array}{c} I(t)=I(0)\exp\left(\int_{0}^{t}(\eta (D(s))-\mu R(s)-(\theta +\tau ))\;ds\right)\geq 0. \end{array}$$

Similarly,


$$\begin{array}{c} R(t)=R(0)\exp\left(\int_{0}^{t}(\rho I(s)-\lambda )\;ds\right)\geq 0. \end{array}$$

Finally,


$$\begin{array}{c} \dot{N}=-\varepsilon \gamma DN, \end{array}$$

so


$$\begin{array}{c} N(t)=N(0)\exp\left(-\varepsilon \gamma \int_{0}^{t}D(s)\;ds\right)\geq 0. \end{array}$$

Therefore, all components remain nonnegative for all t∈[0,T).

### 2.5 Uniform boundedness of solutions

**Theorem 2.** *All solutions of system* (1)–(4) *with nonnegative initial data remain uniformly bounded.*

*Proof.* We establish bounds for each state variable.

First, from the myelin equation,


$$\begin{array}{c} \dot{M}=\varepsilon (\alpha -\beta IM-\delta M)\leq \varepsilon (\alpha -\delta M), \end{array}$$

since I≥0. By the comparison principle,


$$\begin{array}{c} M(t)\leq M(0)e^{-\varepsilon \delta t}+\frac{\alpha }{\delta }\left(1-e^{-\varepsilon \delta t}\right). \end{array}$$

Hence


$$\begin{array}{c} 0\leq M(t)\leq \max\left\{M(0),\frac{\alpha }{\delta }\right\}. \end{array}$$

Next, observe that


$$\begin{array}{c} \eta (D)=\eta_{\max}\frac{D}{\omega +D},\;D\geq 0, \end{array}$$

satisfies


$$\begin{array}{c} 0\leq \eta (D)\leq \eta_{\max}. \end{array}$$

Therefore,


$$\begin{array}{c} \dot{I}=(\eta (D)-(\theta +\tau )-\mu R)I\leq (\eta_{\max}-(\theta +\tau )-\mu R)I, \end{array}$$

while


$$\begin{array}{c} \dot{R}=(\rho I-\lambda )R. \end{array}$$

We distinguish two cases.

**Case 1:** $\eta_{\max}\leq \theta +\tau .$

In this case,


$$\begin{array}{c} \dot{I}\leq -(\theta +\tau -\eta_{\max})I, \end{array}$$

and therefore Let


$$\begin{array}{c} k=\theta +\tau -\eta_{\max}>0. \end{array}$$

Then


$$\begin{array}{c} I(t)\leq I(0)e^{-kt}. \end{array}$$

Hence *I*(*t*) is bounded and converges exponentially to zero.

For *R*(*t*), we write


$$\begin{array}{c} \dot{R}=(\rho I(t)-\lambda )R. \end{array}$$

Solving this scalar equation gives


$$\begin{array}{c} R(t)=R(0)\exp\left(\int_{0}^{t}(\rho I(s)-\lambda )\;ds\right). \end{array}$$

Using the bound on *I*(*t*), we obtain


$$\begin{array}{c} \int_{0}^{t}I(s)\;ds\leq I(0)\int_{0}^{t}e^{-ks}\;ds=\frac{I(0)}{k}\left(1-e^{-kt}\right)\leq \frac{I(0)}{k}. \end{array}$$

Therefore,


$$\begin{array}{c} R(t)\leq R(0)\exp\left(\frac{\rho I(0)}{k}\right). \end{array}$$

Thus *R*(*t*) remains uniformly bounded on [0,∞). Consequently, both immune populations are bounded.

**Case 2:** $\eta_{\max}>\theta +\tau .$

Consider the Lotka–Volterra comparison system


$$\begin{array}{c} \dot{I}=(\eta_{\max}-(\theta +\tau )-\mu R)I,\;\dot{R}=(\rho I-\lambda )R. \end{array}$$

This system admits the positive equilibrium


$$\begin{array}{c} I_{i}=\frac{\lambda }{\rho },\;R_{i}=\frac{\eta_{\max}-(\theta +\tau )}{\mu }. \end{array}$$

Moreover,


$$\begin{array}{c} V(I,R)=\rho I-\lambda \ln I+\mu R-(\eta_{\max}-(\theta +\tau ))\ln R \end{array}$$

is a first integral satisfying


$$\begin{array}{c} \frac{dV}{dt}=0. \end{array}$$

Furthermore,


$$\begin{array}{c} V(I,R)\to \infty \end{array}$$

as


$$\begin{array}{c} I\to 0^{+},\;R\to 0^{+},\;I\to \infty ,\;R\to \infty . \end{array}$$

Hence every level set of *V* is compact in the positive quadrant. Let


$$\begin{array}{c} V_{0}=V(I(0),R(0)) \end{array}$$

and define


$$\begin{array}{c} \Gamma_{V_{0}}=\left\{(I,R)\in {\mathbb{R}}_{+}^{2}:V(I,R)\leq V_{0}\right\}. \end{array}$$

Since $\Gamma_{V_{0}}$ is compact and the function


$$\begin{array}{c} \Phi (I,R)=I+R \end{array}$$

is continuous, there exists


$$\begin{array}{c} \Phi_{max}=\max_{(I,R)\in \Gamma_{V_{0}}}(I+R). \end{array}$$

Therefore,


$$\begin{array}{c} 0\leq I(t)+R(t)\leq \Phi_{max},\;t\geq 0, \end{array}$$

where


$$\begin{array}{c} \Phi_{max}=\Phi_{max}(I(0),R(0),\rho ,\lambda ,\mu ,\eta_{\max},\theta ,\tau )>0. \end{array}$$

Since the original immune subsystem is dominated by the above comparison system, its trajectories remain bounded as well.

Finally, for the neuronal capacity variable,


$$\begin{array}{c} \dot{N}=-\varepsilon \gamma DN, \end{array}$$

with D≥0. Therefore


$$\begin{array}{c} \dot{N}\leq 0, \end{array}$$

which implies


$$\begin{array}{c} 0\leq N(t)\leq N(0),\;t\geq 0. \end{array}$$

Combining the bounds for *M*, *I*, *R*, and *N*, we conclude that all solutions remain uniformly bounded for all t≥0.□

**Corollary 1.** *Under the assumptions of Theorems 1 and 2, every solution of system* (1)–(4) *with nonnegative initial data remains in the region*


$$\begin{array}{c} \Omega =\left\{(M,I,R,N)\in {\mathbb{R}}_{+}^{4}:0\leq M\leq M_{\max}^{*},\;0\leq I+R\leq \Phi^{*},\;0\leq N\leq N(0)\right\}, \end{array}$$

*where*


$$\begin{array}{c} M_{\max}^{*}=\max\left\{M(0),\frac{\alpha }{\delta }\right\}, \end{array}$$

*and*


$$\begin{array}{c} \Phi^{*}=\max\left\{\Phi_{\max},\;I(0)+R(0)\exp\left(\frac{\rho I(0)}{\theta +\tau -\eta_{\max}}\right)\right\}. \end{array}$$

*Consequently,* Ω *is a positively invariant and bounded region for system* (1)–(4).

## 3 Fast immune dynamics

For the analysis of the fast immune dynamics, the neuronal variable *N* is omitted because it is decoupled from the (*M*,*I*,*R*) subsystem and therefore does not affect its equilibrium or stability properties. The variable *N*(*t*) is retained in the full model and in the numerical simulations.

The parameter 0<ε≪1 separates the slow myelin dynamics from the faster immune dynamics. We justify freezing *M* on every fixed fast-time interval 0≤t≤T.

By Corollary 1,


$$\begin{array}{c} 0\leq M(t)\leq M_{\max}^{*},\;0\leq I(t)\leq I(t)+R(t)\leq \Phi^{*}. \end{array}$$

Hence, from


$$\begin{array}{c} \dot{M}=\varepsilon (\alpha -\beta IM-\delta M), \end{array}$$

we obtain


$$\begin{array}{cc} \left|M(t)-M(0)\right| & \leq \varepsilon \int_{0}^{t}\left(\alpha +\beta I(s)M(s)+\delta M(s)\right)\;ds\\ & \leq \varepsilon t\left[\alpha +(\beta \Phi^{*}+\delta )M_{\max}^{*}\right]\\ & \leq \varepsilon T\left[\alpha +(\beta \Phi^{*}+\delta )M_{\max}^{*}\right]. \end{array}$$ (5)

Therefore,


$$\begin{array}{c} M(t)=M(0)+O(\varepsilon ) \end{array}$$

uniformly for 0≤t≤T.

Since


$$\begin{array}{c} D(t)=M_{\max}-M(t), \end{array}$$

it follows that


$$\begin{array}{c} D(t)=D(0)+O(\varepsilon ). \end{array}$$

Moreover,


$$\begin{array}{c} \eta (D)=\eta_{\max}\frac{D}{\omega +D},\;\eta^{\prime }(D)=\frac{\eta_{\max}\omega }{(\omega +D{)}^{2}}\leq \frac{\eta_{\max}}{\omega },\;D\geq 0. \end{array}$$

Thus, using (5),


$$\begin{array}{cc} \left|\eta (D(t))-\eta (D(0))\right| & \leq \frac{\eta_{\max}}{\omega }|D(t)-D(0)|\\ & =\frac{\eta_{\max}}{\omega }|M(t)-M(0)|\\ & \leq \varepsilon \frac{\eta_{\max}}{\omega }T\left[\alpha +(\beta \Phi^{*}+\delta )M_{\max}^{*}\right]. \end{array}$$ (6)

To compare the full and frozen-myelin immune dynamics, define


$$\begin{array}{c} X_{\varepsilon }(t)=(\begin{array}{c} I_{\varepsilon }(t)\\ R_{\varepsilon }(t) \end{array}),\;X_{0}(t)=(\begin{array}{c} I_{0}(t)\\ R_{0}(t) \end{array}), \end{array}$$

and


$$\begin{array}{c} F(X,M)=(\begin{array}{c} \left[\eta (M_{\max}-M)-\mu R-\theta -\tau \right]I\\ (\rho I-\lambda )R \end{array}). \end{array}$$

Then


$$\begin{array}{c} {\dot{X}}_{\varepsilon }=F(X_{\varepsilon },M(t)),\;{\dot{X}}_{0}=F(X_{0},M(0)), \end{array}$$

with


$$\begin{array}{c} X_{\varepsilon }(0)=X_{0}(0). \end{array}$$

For the same immune state $X=(I,R{)}^{⊤}$,


$$\begin{array}{c} F(X,M(t))-F(X,M(0))=(\begin{array}{c} {}[\eta (D(t))-\eta (D(0))]I\\ 0 \end{array}). \end{array}$$

Using $I\leq \Phi^{*}$ and (6),


$$\begin{array}{cc} \left\|F(X,M(t))-F(X,M(0))\right\| & \leq \Phi^{*}|\eta (D(t))-\eta (D(0))|\\ & \leq \varepsilon K_{T}, \end{array}$$ (7)

where


$$\begin{array}{c} K_{T}=\Phi^{*}\frac{\eta_{\max}}{\omega }T\left[\alpha +(\beta \Phi^{*}+\delta )M_{\max}^{*}\right]. \end{array}$$

Since *F* is locally Lipschitz in *X* on the bounded invariant region Ω, there exists $L_{T}>0$ such that


$$\begin{array}{c} \|X_{\varepsilon }(t)-X_{0}(t)\|\leq L_{T}\int_{0}^{t}\|X_{\varepsilon }(s)-X_{0}(s)\|\;ds+\varepsilon K_{T}t. \end{array}$$

Grönwall’s inequality therefore gives


$$\begin{array}{c} \|X_{\varepsilon }(t)-X_{0}(t)\|\leq \varepsilon \frac{K_{T}}{L_{T}}\left(e^{L_{T}t}-1\right), \end{array}$$

and consequently


$$\begin{array}{c} \underset{0\leq t\leq T}{\sup}\|X_{\varepsilon }(t)-X_{0}(t)\|=O(\varepsilon ). \end{array}$$

Thus, on every fixed *O*(1) time interval, the full immune dynamics converge, as ε→0, to the system obtained by freezing the myelin level. Let


$$\begin{array}{c} D_{0}=M_{\max}-M(0). \end{array}$$

The corresponding fast layer problem is


$$\begin{array}{cc} \frac{dI}{dt} & =\left(\eta (D_{0})-\mu R-\theta -\tau \right)I,\\ \frac{dR}{dt} & =\left(\rho I-\lambda \right)R. \end{array}$$ (8)

For notational simplicity, the prescribed damage level *D*_0_ will henceforth be denoted by *D*. Therefore, (8) becomes


$$\begin{array}{c} \frac{dI}{dt}=\left(\eta (D)-\mu R-\theta -\tau \right)I,\;\frac{dR}{dt}=\left(\rho I-\lambda \right)R, \end{array}$$

with *D* treated as a parameter on the fast time scale.

System (8) is the fast layer problem associated with the full slow-fast system, rather than a conventional quasi-steady-state approximation in which the fast variables are assumed to have reached equilibrium.

In particular, as shown below, when


$$\begin{array}{c} \eta (D)>\theta +\tau , \end{array}$$

the positive equilibrium of the fast subsystem is a nonlinear center and is not normally hyperbolic. Thus, the analysis does not require persistence of a normally hyperbolic slow manifold in this regime.

The equilibria and stability properties of (8) are investigated below.

### 3.1 Equilibria of the fast immune subsystem

The equilibria of system (8) satisfy


$$\begin{array}{c} I\left(\eta (D)-\mu R-\theta -\tau \right)=0, \end{array}$$

and


$$\begin{array}{c} R\left(\rho I-\lambda \right)=0. \end{array}$$

The immune-free equilibrium is


$$\begin{array}{c} E_{0}=(0,0). \end{array}$$

For a positive interior equilibrium $E_{i}$,


$$\begin{array}{c} I_{i}=\frac{\lambda }{\rho }, \end{array}$$

and


$$\begin{array}{c} R_{i}=\frac{\eta (D)-\theta -\tau }{\mu }. \end{array}$$

Hence, the immune subsystem admits the interior equilibrium


$$\begin{array}{c} E_{i}=\left(\frac{\lambda }{\rho },\frac{\eta (D)-\theta -\tau }{\mu }\right), \end{array}$$

provided that


$$\begin{array}{c} \eta (D)>\theta +\tau . \end{array}$$

This condition ensures that immune activation exceeds the total removal rate of autoreactive immune cells, allowing the coexistence of autoreactive and regulatory immune cell populations.

At the critical value


$$\begin{array}{c} \eta (D)=\theta +\tau , \end{array}$$

the interior equilibrium reduces to the immune-only boundary equilibrium


$$\begin{array}{c} E_{b}=\left(\frac{\lambda }{\rho },0\right). \end{array}$$

### 3.2 Local stability analysis

Let


$$\begin{array}{c} F_{1}(I,R)=\left(\eta (D)-\mu R-\theta -\tau \right)I, \end{array}$$ (9)

and


$$\begin{array}{c} F_{2}(I,R)=(\rho I-\lambda )R. \end{array}$$ (10)

The Jacobian matrix of system (8) is


$$\begin{array}{c} J(I,R)=(\begin{array}{cc} \eta (D)-\mu R-\theta -\tau & -\mu I\\ \rho R & \rho I-\lambda \end{array}). \end{array}$$ (11)

#### 3.2.1 Immune-free equilibrium.

The immune-free equilibrium of the immune subsystem is


$$\begin{array}{c} E_{0}=(0,0). \end{array}$$

The Jacobian matrix evaluated at *E*_0_ is


$$\begin{array}{c} J(E_{0})=(\begin{array}{cc} \eta (D)-\theta -\tau & 0\\ 0 & -\lambda \end{array}). \end{array}$$ (12)

Hence, the corresponding eigenvalues are


$$\begin{array}{c} \xi_{1}=\eta (D)-\theta -\tau ,\;\xi_{2}=-\lambda . \end{array}$$ (13)

Therefore, the immune-free equilibrium is locally asymptotically stable whenever


$$\begin{array}{c} \eta (D)<\theta +\tau , \end{array}$$ (14)

and unstable whenever


$$\begin{array}{c} \eta (D)>\theta +\tau . \end{array}$$ (15)

Motivated by this threshold condition, we define the damage-dependent autoimmune activation number


$$\begin{array}{c} R_{0}=\frac{\eta (D)}{\theta +\tau }=\frac{\eta_{\max}}{\theta +\tau }\frac{D}{\omega +D}. \end{array}$$ (16)

Using $D=M_{\max}-M$, this may also be written as


$$\begin{array}{c} R_{0}=\frac{\eta_{\max}}{\theta +\tau }\frac{M_{\max}-M}{\omega +M_{\max}-M}. \end{array}$$

Consequently,


$$\begin{array}{c} R_{0}<1\;⟺\;\eta (D)<\theta +\tau , \end{array}$$

whereas


$$\begin{array}{c} R_{0}>1\;⟺\;\eta (D)>\theta +\tau . \end{array}$$

The quantity $R_{0}$ compares the activation of autoreactive immune cells induced by tissue damage with their overall removal rate. It therefore measures the ability of a given level of myelin damage to promote autoimmune activity. When $R_{0}<1$, immune removal dominates damage-induced activation and autoreactive immune activity declines. In contrast, when $R_{0}>1$, damage-induced activation exceeds immune removal, causing autoreactive immune activity to increase. Consequently, $R_{0}=1$ represents the threshold separating immune control from immune activation.

The threshold condition may also be expressed in terms of the treatment parameter. Defining


$$\begin{array}{c} \tau_{c}=\eta (D)-\theta , \end{array}$$ (17)

we obtain


$$\begin{array}{c} \tau >\tau_{c}\;⟺\;R_{0}<1, \end{array}$$

and


$$\begin{array}{c} \tau <\tau_{c}\;⟺\;R_{0}>1. \end{array}$$

Hence, $\tau_{c}$ represents the critical treatment level required to suppress autoreactive immune activation for a given level of myelin damage. Since η(D) is an increasing function of *D*, larger levels of tissue damage require stronger treatment to maintain immune control. This highlights the central role of myelin damage in determining both disease progression and treatment effectiveness.

#### 3.2.2 Interior equilibrium.

Suppose that


$$\begin{array}{c} \eta (D)>\theta +\tau , \end{array}$$ (18)

so that the interior equilibrium


$$\begin{array}{c} E_{i}=\left(\frac{\lambda }{\rho },\frac{\eta (D)-\theta -\tau }{\mu }\right) \end{array}$$ (19)

exists.

Evaluating the Jacobian at $E_{i}$ gives


$$\begin{array}{c} J(E_{i})=(\begin{array}{cc} 0 & -\mu \lambda /\rho \\ \rho (\eta (D)-\theta -\tau )/\mu & 0 \end{array}). \end{array}$$ (20)

The characteristic equation is


$$\begin{array}{c} \xi^{2}+\lambda (\eta (D)-\theta -\tau )=0. \end{array}$$ (21)

Thus,


$$\begin{array}{c} \xi_{1,2}=\pm i\sqrt{\lambda (\eta (D)-\theta -\tau )}. \end{array}$$ (22)

Therefore, whenever the interior equilibrium exists, its eigenvalues are purely imaginary. Consequently, the linearization predicts a center and linear stability analysis alone is inconclusive.

**Nonlinear behavior of the interior equilibrium**

Assume that


$$\begin{array}{c} \eta (D)>\theta +\tau . \end{array}$$

Define


$$\begin{array}{c} a=\eta (D)-\theta -\tau >0. \end{array}$$

Then the immune subsystem becomes


$$\begin{array}{c} \frac{dI}{dt}=(a-\mu R)I,\;\frac{dR}{dt}=(\rho I-\lambda )R. \end{array}$$ (23)

The interior equilibrium is


$$\begin{array}{c} E_{i}=\left(\frac{\lambda }{\rho },\frac{a}{\mu }\right). \end{array}$$

We now show that $E_{i}$ is a nonlinear center.

Consider the function


$$\begin{array}{c} H(I,R)=\rho I-\lambda \ln I+\mu R-a\ln R,\;I>0,\;R>0. \end{array}$$ (24)

Differentiating *H* along solutions gives


$$\begin{array}{c} \frac{dH}{dt}=\left(\rho -\frac{\lambda }{I}\right)\frac{dI}{dt}+\left(\mu -\frac{a}{R}\right)\frac{dR}{dt}. \end{array}$$

Substituting the system equations yields


$$\begin{array}{c} \frac{dH}{dt}=\left(\rho -\frac{\lambda }{I}\right)(a-\mu R)I+\left(\mu -\frac{a}{R}\right)(\rho I-\lambda )R. \end{array}$$

Since


$$\begin{array}{c} \left(\rho -\frac{\lambda }{I}\right)I=\rho I-\lambda , \end{array}$$

and


$$\begin{array}{c} \left(\mu -\frac{a}{R}\right)R=\mu R-a, \end{array}$$

we obtain


$$\begin{array}{c} \frac{dH}{dt}=(\rho I-\lambda )(a-\mu R)+(\mu R-a)(\rho I-\lambda ). \end{array}$$

Using


$$\begin{array}{c} \mu R-a=-(a-\mu R), \end{array}$$

it follows that


$$\begin{array}{c} \frac{dH}{dt}=(\rho I-\lambda )(a-\mu R)-(a-\mu R)(\rho I-\lambda )=0. \end{array}$$

Hence,


$$\begin{array}{c} H(I,R)=\text{constant} \end{array}$$

along every solution trajectory.

Next, compute the first derivatives:


$$\begin{array}{c} H_{I}=\rho -\frac{\lambda }{I},\;H_{R}=\mu -\frac{a}{R}. \end{array}$$

Therefore,


$$\begin{array}{c} H_{I}=0\;⟺\;I=\frac{\lambda }{\rho }, \end{array}$$

and


$$\begin{array}{c} H_{R}=0\;⟺\;R=\frac{a}{\mu }. \end{array}$$

Thus, the only critical point of *H* in the positive quadrant is $E_{i}$.

The second derivatives are


$$\begin{array}{c} H_{II}=\frac{\lambda }{I^{2}}>0,\;H_{RR}=\frac{a}{R^{2}}>0,\;H_{IR}=0. \end{array}$$

Hence, the Hessian matrix is


$$\begin{array}{c} \nabla^{2}H=(\begin{array}{cc} \lambda /I^{2} & 0\\ 0 & a/R^{2} \end{array}), \end{array}$$

which is positive definite throughout the positive quadrant.

Therefore, *H* has a strict local minimum at $E_{i}$.

Furthermore,


$$\begin{array}{c} H(I,R)\to \infty \end{array}$$

as


$$\begin{array}{c} I\to 0^{+},\;R\to 0^{+},\;I\to \infty ,\;R\to \infty . \end{array}$$

Consequently, every level set


$$\begin{array}{c} H(I,R)=C,\;C>H(E_{i}), \end{array}$$

sufficiently close to $E_{i}$ is a simple closed curve surrounding $E_{i}$.

Since *H* is conserved along trajectories, solutions starting on one of these level curves remain on that curve for all time. Therefore, trajectories sufficiently close to $E_{i}$ are closed orbits surrounding $E_{i}$.

We conclude that the interior equilibrium


$$\begin{array}{c} E_{i}=\left(\frac{\lambda }{\rho },\frac{\eta (D)-\theta -\tau }{\mu }\right) \end{array}$$

is a nonlinear center.

#### 3.2.3 Immune-only boundary equilibrium.

At the critical value


$$\begin{array}{c} \eta (D)=\theta +\tau , \end{array}$$

the interior equilibrium reduces to the immune-only boundary equilibrium


$$\begin{array}{c} E_{b}=\left(\frac{\lambda }{\rho },0\right). \end{array}$$

Evaluating the Jacobian at $E_{b}$ yields


$$\begin{array}{c} J(E_{b})=(\begin{array}{cc} 0 & -\mu \lambda /\rho \\ 0 & 0 \end{array}), \end{array}$$

whose characteristic equation is


$$\begin{array}{c} \xi^{2}=0. \end{array}$$

Hence, the point


$$\begin{array}{c} E_{b}=\left(\frac{\lambda }{\rho },0\right) \end{array}$$

possesses a double zero eigenvalue, and linearization is therefore inconclusive. This is consistent with the degenerate critical structure described above at


$$\begin{array}{c} \eta (D)=\theta +\tau . \end{array}$$

### 3.3 Global stability of the immune-free equilibrium

**Theorem 3.** *If*


$$\begin{array}{c} R_{0}<1, \end{array}$$

*then the immune-free equilibrium*


$$\begin{array}{c} E_{0}=(0,0) \end{array}$$

*of system* (8) *is globally asymptotically stable in* ${\mathbb{R}}_{+}^{2}$.

*Proof.* The condition $R_{0}<1$ is equivalent to


$$\begin{array}{c} \eta (D)<\theta +\tau . \end{array}$$

Let


$$\begin{array}{c} c=\theta +\tau -\eta (D)>0. \end{array}$$

Consider the Lyapunov function


$$\begin{array}{c} V(I,R)=I+\frac{\mu }{\rho }R. \end{array}$$

Clearly,


$$\begin{array}{c} V(I,R)\geq 0 \end{array}$$

for all $(I,R)\in {\mathbb{R}}_{+}^{2}$, and


$$\begin{array}{c} V(I,R)=0\;⟺\;(I,R)=(0,0). \end{array}$$

Differentiating *V* along solutions gives


$$\begin{array}{c} \frac{dV}{dt}=\frac{dI}{dt}+\frac{\mu }{\rho }\frac{dR}{dt}. \end{array}$$

Using system (8), we obtain


$$\begin{array}{c} \frac{dV}{dt}=\left(\eta (D)-\mu R-\theta -\tau \right)I+\frac{\mu }{\rho }(\rho I-\lambda )R. \end{array}$$

Expanding,


$$\begin{array}{c} \frac{dV}{dt}=(\eta (D)-\theta -\tau )I-\mu IR+\mu IR-\frac{\mu \lambda }{\rho }R. \end{array}$$

Thus,


$$\begin{array}{c} \frac{dV}{dt}=(\eta (D)-\theta -\tau )I-\frac{\mu \lambda }{\rho }R. \end{array}$$

Since


$$\begin{array}{c} \eta (D)-\theta -\tau =-c<0, \end{array}$$

we get


$$\begin{array}{c} \frac{dV}{dt}=-cI-\frac{\mu \lambda }{\rho }R\leq 0. \end{array}$$

Moreover.

Therefore, by Lyapunov’s direct method, the immune-free equilibrium *E*_0_ = (0,0) is globally asymptotically stable in ${\mathbb{R}}_{+}^{2}$.

### 3.4 Treatment-induced stability and long-term dynamics

The threshold quantity


$$\begin{array}{c} R_{0}(M)=\frac{\eta (M_{\max}-M)}{\theta +\tau } \end{array}$$

depends on the current myelin level *M*. Since


$$\begin{array}{c} D=M_{\max}-M, \end{array}$$

and η(D) is an increasing function of the damage level *D*, it follows that $R_{0}(M)$ is a continuous decreasing function of *M*. Consequently, there exists a unique critical myelin level $M_{c}$ satisfying


$$\begin{array}{c} R_{0}(M_{c})=1. \end{array}$$

The slow myelin dynamics provide a biological interpretation of the treatment-induced regimes. When the treatment level exceeds the critical threshold $\tau_{c}$, the immune-free equilibrium *E*_0_ is globally asymptotically stable and the immune populations converge to zero. In this case, the slow myelin equation reduces to


$$\begin{array}{c} \dot{M}=\alpha -\delta M, \end{array}$$

yielding the disease-free myelin level


$$\begin{array}{c} M_{0}=\frac{\alpha }{\delta }. \end{array}$$

When the treatment level is below $\tau_{c}$, the interior equilibrium $E_{i}$ exists and the immune subsystem exhibits sustained oscillatory dynamics around the representative autoreactive burden


$$\begin{array}{c} I_{i}=\frac{\lambda }{\rho }. \end{array}$$

Substituting this value into the slow myelin equation gives


$$\begin{array}{c} \dot{M}=\alpha -\delta M-\beta M\frac{\lambda }{\rho }, \end{array}$$

which yields the inflammatory myelin level


$$\begin{array}{c} M_{i}=\frac{\alpha }{\delta +\beta \lambda /\rho }. \end{array}$$

Since


$$\begin{array}{c} \delta +\frac{\beta \lambda }{\rho }>\delta , \end{array}$$

we obtain


$$\begin{array}{c} M_{i}<M_{0}. \end{array}$$

For the parameter regime considered in this work, the disease-free state satisfies


$$\begin{array}{c} R_{0}(M_{0})<1, \end{array}$$

whereas the inflammatory state satisfies


$$\begin{array}{c} R_{0}(M_{i})>1. \end{array}$$

Since $R_{0}(M)$ is continuous and strictly decreasing in *M*, the unique threshold level $M_{c}$ must lie between $M_{i}$ and *M*_0_. Therefore,


$$\begin{array}{c} 0\leq M_{i}<M_{c}<M_{0}\leq M_{\max}. \end{array}$$

Defining the corresponding damage levels by


$$\begin{array}{c} D_{j}=M_{\max}-M_{j},\;j=i,c,0, \end{array}$$

gives


$$\begin{array}{c} D_{i}>D_{c}>D_{0}. \end{array}$$

Associated with these damage levels are the treatment values


$$\begin{array}{c} \tau_{i}=\eta (D_{i})-\theta ,\;\tau_{c}=\eta (D_{c})-\theta ,\;\tau_{0}=\eta (D_{0})-\theta . \end{array}$$

Since η(D) is increasing in *D*, it follows that


$$\begin{array}{c} \tau_{i}>\tau_{c}>\tau_{0}. \end{array}$$

The threshold $\tau_{c}$ is the only critical treatment value governing the transition between immune persistence and immune suppression. The quantities $\tau_{i}$ and $\tau_{0}$ are included only as reference values associated with the representative inflammatory and disease-free myelin states.

Fig 2 summarizes the treatment-dependent stability and threshold structure of the reduced immune subsystem. For treatment levels below the critical threshold $\tau_{c}$, the immune-free equilibrium *E*_0_ is unstable, while the interior equilibrium $E_{i}$ exists as a nonlinear center surrounded by closed periodic orbits. At the critical threshold $\tau =\tau_{c}$ (equivalently, ${ℛ}_{0}=1$), the system possesses the continuum of boundary equilibria


$$\begin{array}{c} {ℰ}_{c}=\{(I,0):I\geq 0\}, \end{array}$$

with


$$\begin{array}{c} E_{b}=\left(\frac{\lambda }{\rho },0\right) \end{array}$$

being the limiting position of $E_{i}$ as $\tau \to \tau_{c}^{-}$. For treatment levels above $\tau_{c}$, the interior equilibrium no longer exists and the immune-free equilibrium *E*_0_ is globally asymptotically stable. Thus, $\tau_{c}$ represents the critical treatment threshold separating persistent autoimmune oscillations from immune suppression.

## 4 Optimal control with smooth treatment profiles

To investigate treatment strategies that reduce autoimmune activity while accounting for treatment intensity and its rate of adjustment, we formulate an optimal control problem over a fixed treatment horizon [0,*T*].

The constant treatment parameter τ considered in the preceding sections is replaced by a time-dependent treatment intensity *u*(*t*). We introduce the control variable $a(t)=\dot{u}(t)$, represen*t*ing the rate of treatment adjustment. Accordingly, *u*(*t*) is treated as an additional state variable, while *a*(*t*) is the con*t*rol to be optimized.

Positive values of *a*(*t*) correspond to treatment escalation, whereas negative values represent treatment reduction. Since $a\in L^{\infty }(0,T)$ and


$$\begin{array}{c} u(t)=u_{0}+\int_{0}^{t}a(s)\;ds, \end{array}$$

the treatment intensity satisfies $u\in W^{1,\infty }(0,T)$ and is therefore Lipschitz continuous.

### Controlled system and objective functional

The controlled system is given by


$$\begin{array}{cc} \dot{M}(t) & =\varepsilon (\alpha -\beta I(t)M(t)-\delta M(t)),\\ \dot{I}(t) & =(\eta (D(M(t)))-\mu R(t)-\theta -u(t))I(t),\\ \dot{R}(t) & =(\rho I(t)-\lambda )R(t),\\ \dot{u}(t) & =a(t), \end{array}$$ (25)

subject to


$$\begin{array}{c} M(0)=M_{0},\;I(0)=I_{0},\;R(0)=R_{0},\;u(0)=u_{0}. \end{array}$$ (26)

The objective functional is defined by


$$\begin{array}{c} J(a)=\int_{0}^{T}\left[AI(t)+Ba(t{)}^{2}+Cu(t)\right]dt+q_{I}I(T), \end{array}$$ (27)

where


$$\begin{array}{c} A>0,\;B>0,\;C>0,\;q_{I}\geq 0. \end{array}$$

The term *AI*(*t*) penalizes autoreactive immune-cell burden, while *Ba*(*t*)^2^ penalizes rapid changes in treatment intensity. The terminal term $q_{I}I(T)$ penalizes residual autoimmune activity at the end of the treatment horizon.

The additional linear term *Cu*(*t*) penalizes cumulative treatment exposure. Thus, the objective functional balances autoimmune suppression, treatment intensity, and the rate of treatment adjustment.

The admissible control set is defined by


$$\begin{array}{cc} U=\{a\in L^{\infty }(0,T): & \;a_{\min}\leq a(t)\leq a_{\max}\;\text{a.e.},\\ & \;0\leq u_{0}+\int_{0}^{t}a(s)\;ds\leq u_{\max},\;t\in [0,T]\}, \end{array}$$ (28)

where


$$\begin{array}{c} a_{\min}\leq 0\leq a_{\max},\;0\leq u_{0}\leq u_{\max}. \end{array}$$

The optimal control problem consists of finding $a^{*}\in U$ satisfying


$$\begin{array}{c} J(a^{*})=\min_{a\in U}J(a). \end{array}$$ (29)

### Existence of an optimal control

**Theorem 4.** *Assume that the biological parameters are positive, the initial conditions are nonnegative, and*


$$\begin{array}{c} 0\leq M_{0}\leq M_{\max},\;\frac{\alpha }{\delta }\leq M_{\max}. \end{array}$$

*Then the optimal control problem admits at least one optimal control* $a^{*}\in U$.

**Proof.** The admissible set U is nonempty because *a*(*t*) = 0 is admissible for any $u_{0}\in [0,u_{\max}]$.

The positivity and boundedness analysis established earlier provides the basis for the corresponding properties of the controlled system. In particular, since u(t)≥0,


$$\begin{array}{c} 0\leq M(t)\leq \max\left\{M_{0},\frac{\alpha }{\delta }\right\}\leq M_{\max}, \end{array}$$

and


$$\begin{array}{c} 0\leq I(t)\leq I_{0}e^{\eta_{\max}T}. \end{array}$$

Furthermore,


$$\begin{array}{c} 0\leq R(t)\leq R_{0}\exp\left(\rho I_{0}Te^{\eta_{\max}T}\right). \end{array}$$

Thus, the biological state variables remain bounded on the finite interval [0,*T*].

Since the admissible controls are uniformly bounded in $L^{\infty }(0,T)$, any minimizing sequence $\left\{a_{n}\right\}$ admits a subsequence converging weakly in *L*^2^(0,*T*) to some $a^{*}$. The control bounds are preserved under weak convergence because they define a closed convex subset of *L*^2^(0,*T*).

Moreover, the corresponding treatment profiles


$$\begin{array}{c} u_{n}(t)=u_{0}+\int_{0}^{t}a_{n}(s)\;ds \end{array}$$

are uniformly bounded and equi-Lipschitz. By the Arzelà–Ascoli theorem, a subsequence converges uniformly to


$$\begin{array}{c} u^{*}(t)=u_{0}+\int_{0}^{t}a^{*}(s)\;ds, \end{array}$$

which preserves the treatment-state constraints. Consequently, $a^{*}\in U$.

The boundedness of the biological states and the regularity of the state equations imply, along a subsequence, uniform convergence of the associated state trajectories to the solution corresponding to $a^{*}$.

Finally, the running cost


$$\begin{array}{c} L(I,u,a)=AI+Ba^{2}+Cu \end{array}$$

is continuous in the state variables and convex in *a*. The state-dependent terms converge under uniform convergence, while the quadratic control cost is weakly lower semicontinuous. Hence,


$$\begin{array}{c} J(a^{*})\leq \underset{n\to \infty }{lim inf}J(a_{n})=\underset{a\in U}{\inf}J(a). \end{array}$$

Therefore, $a^{*}$ is an optimal control.

### Hamiltonian and adjoint system

Let $\lambda_{M}$, $\lambda_{I}$, $\lambda_{R}$, and $\lambda_{u}$ denote the adjoint variables associated with the states *M*, *I*, *R*, and *u*, respectively.

The Hamiltonian is


$$\begin{array}{cc} H & =AI+Ba^{2}+Cu\\ & \;+\lambda_{M}\varepsilon (\alpha -\beta IM-\delta M)\\ & \;+\lambda_{I}(\eta (D(M))-\mu R-\theta -u)I\\ & \;+\lambda_{R}(\rho I-\lambda )R+\lambda_{u}a. \end{array}$$ (30)

On intervals where the treatment-intensity state constraint is inactive, Pontryagin’s Maximum Principle gives


$$\begin{array}{c} {\dot{\lambda }}_{j}=-\frac{\partial H}{\partial x_{j}},\;x_{j}\in \{M,I,R,u\}. \end{array}$$

Recall that


$$\begin{array}{c} D(M)=M_{\max}-M,\;\eta (D)=\eta_{\max}\frac{D}{\omega +D}. \end{array}$$

Therefore,


$$\begin{array}{c} \eta_{M}(M):=\frac{d\eta (D(M))}{dM}=-\frac{\eta_{\max}\omega }{(\omega +M_{\max}-M{)}^{2}}. \end{array}$$ (31)

The adjoint system is consequently


$$\begin{array}{cc} {\dot{\lambda }}_{M} & =\varepsilon \lambda_{M}(\beta I+\delta )-\lambda_{I}I\eta_{M}(M),\\ {\dot{\lambda }}_{I} & =-A+\varepsilon \beta M\lambda_{M}\\ & \;-\lambda_{I}(\eta (D(M))-\mu R-\theta -u)-\rho R\lambda_{R},\\ {\dot{\lambda }}_{R} & =\mu I\lambda_{I}-(\rho I-\lambda )\lambda_{R},\\ {\dot{\lambda }}_{u} & =\lambda_{I}I-C. \end{array}$$ (32)

The linear treatment-intensity cost modifies the adjoint equation associated with *u* through the term −C. Consequently, although the algebraic optimality condition for *a* remains unchanged, the resulting treatment strategy depends on *C* through the adjoint variable $\lambda_{u}$.

The terminal cost is


$$\begin{array}{c} \Phi =q_{I}I(T). \end{array}$$

If the treatment-intensity constraint is inactive at the terminal time, the transversality conditions are


λM(T)=0,λI(T)=qI,λR(T)=0,λu(T)=0. (33)

### Characterization of the optimal control

On intervals where


$$\begin{array}{c} 0<u^{*}(t)<u_{\max}, \end{array}$$

the Hamiltonian is minimized with respect to the treatment-adjustment rate *a*.

The stationarity condition


$$\begin{array}{c} \frac{\partial H}{\partial a}=2Ba+\lambda_{u}=0 \end{array}$$

gives the unconstrained candidate


$$\begin{array}{c} \hat{a}(t)=-\frac{\lambda_{u}(t)}{2B}. \end{array}$$

Taking into account the adjustment-rate bounds, the optimal control on interior treatment-state arcs is characterized by


a*(t)=Π[amin,amax](−λu(t)2B), (34)

where Π denotes projection onto the admissible interval.

The corresponding treatment intensity satisfies


u*(t)=u0+∫0ta*(s)ds. (35)

### Treatment-state constraints and numerical implementation

Because *u*(*t*) is an additional state variable, the treatment-intensity bounds constitute state constraints. In particular, feasibility requires the boundary-direction conditions


u(t)=0⟹a(t)≥0,u(t)=umax⟹a(t)≤0. (36)

On any interval where the treatment intensity remains at one of these boundaries, *a*(*t*) = 0 almost everywhere.

In the numerical implementation, we employ a viability-projected forward–backward sweep method (FBSM), using the fourth-order Runge–Kutta method for the state and adjoint equations. The treatment-adjustment rate is projected onto a feasible interval at each time step so that the resulting treatment profile respects the prescribed intensity and adjustment-rate bounds.

The treatment-intensity penalty *Cu* is included in both the objective evaluation and the treatment-state adjoint equation. The computed treatment strategy is assessed using convergence diagnostics, constraint violations, and comparisons of the objective functional with the no-treatment baseline.

The projection procedure enforces numerical feasibility of the treatment profile. However, it should be distinguished from a complete state-constrained Pontryagin formulation, which may require additional multipliers and boundary-arc conditions when treatment-intensity constraints are active.

## 5 Numerical simulations and optimal control results

The numerical simulations presented in this section are used to validate the analytical results and to provide a biological interpretation of the model dynamics. In particular, we investigate how the treatment parameter τ influences immune activity, myelin dynamics, and the transition between persistent autoimmune oscillations and immune suppression.

### 5.1 Time series solutions

The analytical results established that the reduced immune subsystem is governed by a critical treatment threshold $\tau_{c}$, which separates two qualitatively distinct regimes. When $\tau <\tau_{c}$, the immune-free equilibrium *E*_0_ is unstable and the interior equilibrium $E_{i}$ exists as a nonlinear center surrounded by closed periodic orbits.

These periodic orbits are a property of the reduced frozen-myelin subsystem. In the full model, where the myelin level evolves slowly, the numerical simulations exhibit recurrent oscillatory immune dynamics that are qualitatively consistent with the reduced-system behavior, but exact periodicity is not asserted.

In this regime, the full model therefore exhibits recurrent autoimmune oscillations associated with repeated inflammatory activity. In contrast, when $\tau >\tau_{c}$, the interior equilibrium $E_{i}$ does not exist and the immune-free equilibrium *E*_0_ becomes globally asymptotically stable, corresponding to suppression of sustained autoimmune activity.

To illustrate these two regimes, we select representative treatment values on either side of the threshold $\tau_{c}$ and examine the corresponding time-series and phase-plane dynamics.

The baseline parameter values and associated units used throughout the simulations are listed in Table 2. Parameter values were selected from the literature whenever available and supplemented by biologically reasonable representative values when direct experimental estimates were unavailable. Consequently, the numerical simulations are intended primarily to illustrate the qualitative behavior of the model and the influence of the principal mechanisms governing immune activation, immune regulation, myelin damage, and treatment response. The numerical results should therefore be interpreted as qualitative and mechanistic insights into the disease dynamics rather than quantitatively calibrated patient-specific predictions.

**Table 2:** Baseline parameter values and units used in the numerical simulations and optimal control experiments.

Parameter	Value	Units
ε	0.05	dimensionless
α	1.0	myelin·time^–1^
β	0.1	${\text{cell}}^{-1}\cdot$time^–1^
δ	0.3	time^–1^
γ	10^–5^	time^–1^
μ	0.5	${\text{cell}}^{-1}\cdot$time^–1^
θ	0.25	time^–1^
τ	variable	time^–1^
ρ	0.25	${\text{cell}}^{-1}\cdot$time^–1^
λ	0.35	time^–1^
$M_{\max}$	40	myelin
$\eta_{\max}$	2.0	time^–1^
ω	2	myelin
*A*	100	$\text{cost}\cdot {\text{cell}}^{-1}\cdot {\text{time}}^{-1}$
*B*	3	$\text{cost}\cdot {\text{time}}^{3}$
$q_{I}$	500	$\text{cost}\cdot {\text{cell}}^{-1}$
$u_{\min}$	0.0	time^–1^
$u_{\max}$	1.0	time^–1^
$a_{\min}$	−1	time^–2^
$a_{\max}$	1	time^–2^

The treatment values associated with the representative myelin states are


$$\begin{array}{c} \tau_{c}=\eta (D_{c})-\theta ,\;\tau_{i}=\eta (D_{i})-\theta ,\;\tau_{0}=\eta (D_{0})-\theta . \end{array}$$

Using the baseline parameter values, we obtain


$$\begin{array}{c} \tau_{i}=1.547235,\;\tau_{i}>\tau_{c}>\tau_{0},\;\tau_{0}=1.535714. \end{array}$$

Thus, $\tau_{c}$ is the critical treatment threshold separating persistent autoimmune activity from immune suppression, while $\tau_{i}$ and $\tau_{0}$ are reference treatment values corresponding to the representative myelin levels $M_{i}$ and *M*_0_, respectively.

Unless otherwise stated, all numerical simulations are performed with the initial condition [(M(0),I(0),R(0),N(0)) = (19, 2, 2, 15).] This initial condition represents a state with substantial healthy myelin together with elevated autoreactive and regulatory immune cell populations, allowing the transient and asymptotic dynamics of the model to be illustrated under different treatment levels.

Representative values of τ are selected from the different regions determined by these treatment levels to illustrate the corresponding model dynamics.

- **Case 1:** τ=0 $\left(\tau <\tau_{c}\right)$. This case corresponds to the autoimmune regime in which $R_{0}>1$.

The corresponding dynamics are illustrated in Fig 3, which combines the myelin, immune-cell, and phase-plane results into three panels.

**Fig 3:**
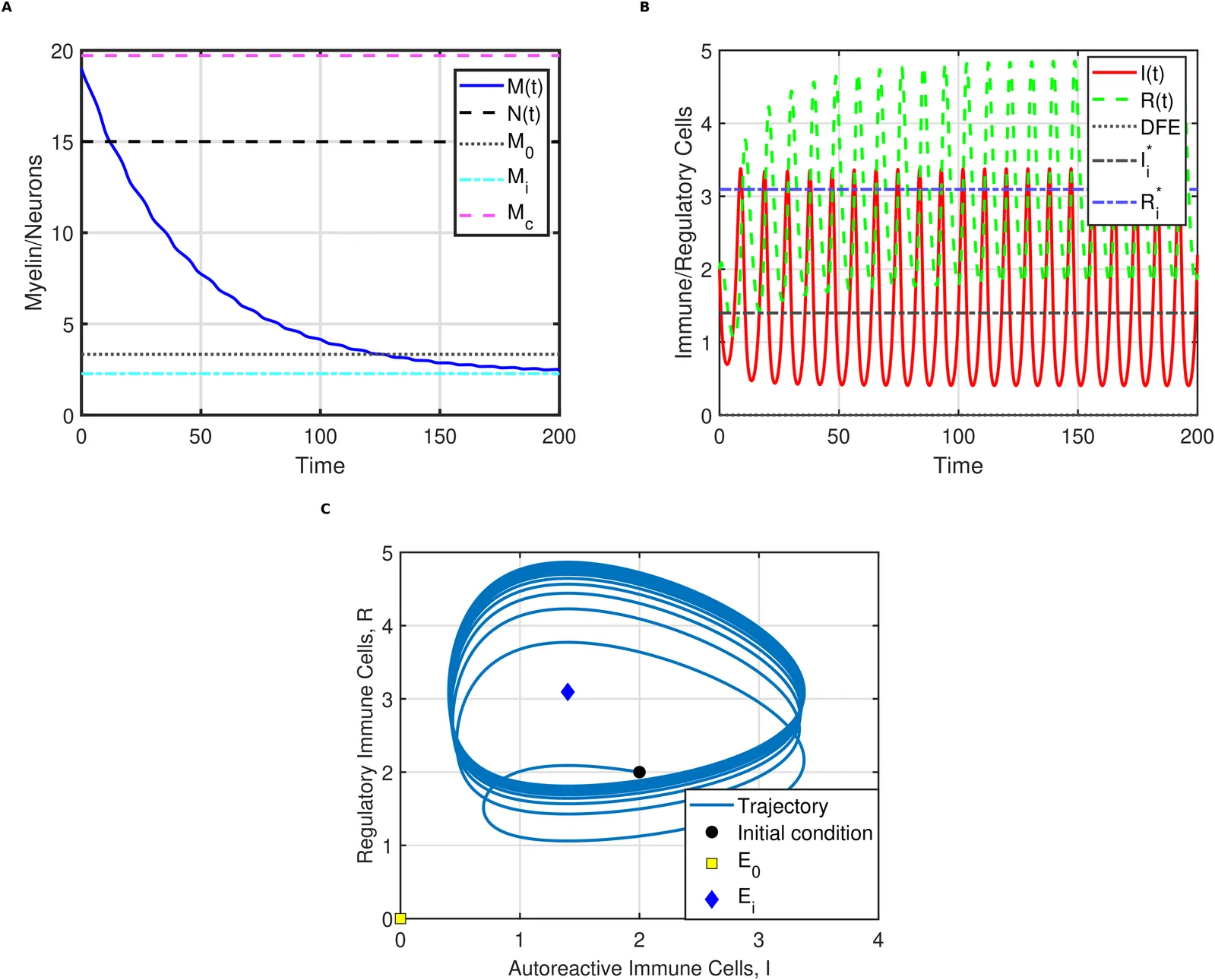
Immune and myelin dynamics for ${ℛ}_{0}>1$. Time-series dynamics for τ=0, corresponding to ${ℛ}_{0}=7.1429>1$. (A) Evolution of healthy myelin *M*(*t*) and neuronal capacity *N*(*t*). (B) Evolution of autoreactive immune cells *I*(*t*) and regulatory immune cells *R*(*t*). (C) Phase-plane trajectory of autoreac*t*ive immune cells *I*(*t*) and regula*t*ory immune cells *R*(*t*).

Panel A shows the evolution of healthy myelin *M*(*t*) and neuronal capacity *N*(*t*). The healthy myelin level decreases over time toward the representative inflammatory myelin level, while neuronal capacity declines as damage accumulates. These dynamics illustrate the effects of persistent autoimmune activity on myelin preservation and neuronal function within the proposed model.

For the parameter values used in the simulation, the representative inflammatory state is


$$\begin{array}{c} (M_{i},I_{i},R_{i})=(2.272727,1.400000,3.094470). \end{array}$$

Here, $\left(I_{i},R_{i}\right)$ denotes the interior equilibrium of the reduced immune subsystem, while $M_{i}$ is the corresponding representative myelin level.

Since $R_{0}>1$, the immune-free equilibrium of the reduced subsystem is unstable, whereas its interior equilibrium is a nonlinear center surrounded by closed periodic orbits. This theoretical result characterizes the reduced frozen-myelin dynamics and does not, by itself, establish exact periodicity in the full model.

Panel B presents the corresponding immune-cell dynamics. The autoreactive immune-cell population *I*(*t*) exhibits recurrent peaks accompanied by oscillations in the regulatory immune-cell population *R*(*t*). This interaction produces repeated episodes of elevated and reduced immune activity, qualitatively representing recurrent inflammatory dynamics within the model.

Panel C shows the (*I*,*R*) phase-plane projection of the full-system trajectory. The recurrent oscillatory structure is qualitatively consistent with the closed-orbit behavior established for the reduced immune subsystem. However, exact periodicity is not asserted for the full system because the myelin level evolves over time.

- **Case 2:** τ=2 $\left(\tau >\tau_{c}\right)$. This case corresponds to the immune-suppression regime in which ${ℛ}_{0}<1$.

The corresponding dynamics are illustrated in Fig 4, which presents the myelin, immune-cell, and phase-plane results in three panels.

**Fig 4:**
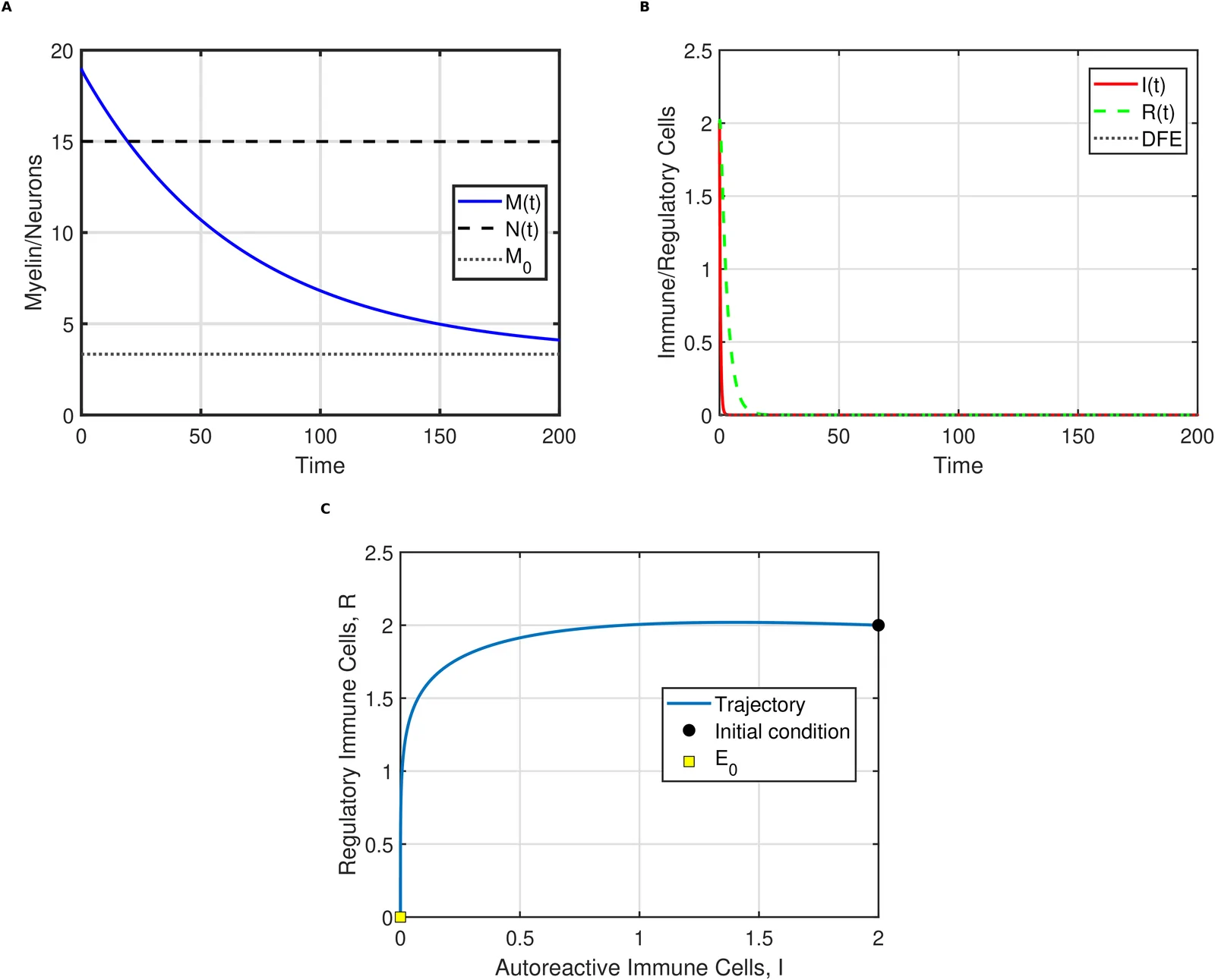
Immune and myelin dynamics for ${ℛ}_{0}<1$. Time-series dynamics for τ=2, corresponding to ${ℛ}_{0}=0.7937<1$. (A) Evolution of healthy myelin *M*(*t*) and neuronal capacity *N*(*t*). (B) Evolution of autoreactive immune cells *I*(*t*) and regulatory immune cells *R*(*t*). (C) Phase-plane trajectory of autoreac*t*ive immune cells *I*(*t*) and regula*t*ory immune cells *R*(*t*).

Panel A shows the evolution of healthy myelin *M*(*t*) and neuronal capacity *N*(*t*). As autoreactive immune activity is suppressed, the healthy myelin level approaches its immune-free equilibrium value. Neuronal capacity decreases during the transient period of myelin deficiency and subsequently changes more slowly as myelin recovers. Since neuronal recovery is not included in the model, the accumulated loss of neuronal capacity is not reversed.

Panel B presents the evolution of autoreactive immune cells *I*(*t*) and regulatory immune cells *R*(*t*). Both populations decrease toward zero, illustrating the suppression of persistent autoimmune activity under the higher treatment level.

Panel C shows the corresponding (*I*,*R*) phase-plane trajectory. The numerical trajectory approaches the immune-free state (*I*,*R*) = (0,0), consistent with the global asymptotic stability established analytically for the reduced immune subsystem when ${ℛ}_{0}<1$.

For the (*M*,*I*,*R*) subsystem, the immune-free equilibrium is


$$\begin{array}{c} (M_{0},I_{0},R_{0})=\left(\frac{\alpha }{\delta },0,0\right)=(3.333333,0,0). \end{array}$$

The neuronal variable *N*(*t*) is not included in this equilibrium representation because it does not feed back into the myelin–immune dynamics.

The numerical simulations of the full model are qualitatively consistent with the analytical results obtained for the reduced immune subsystem. When $\tau <\tau_{c}$, recurrent oscillatory immune dynamics are observed, consistent with the nonlinear-center and closed-orbit structure of the reduced system. In contrast, when $\tau >\tau_{c}$, the numerical trajectories show decreasing autoreactive and regulatory immune-cell populations, together with recovery of the myelin level toward its immune-free equilibrium. These findings illustrate the treatment-dependent transition between recurrent immune activity and immune suppression predicted by the reduced-system analysis, while recognizing that the global stability theorem applies specifically to the reduced immune subsystem.

Fig 5 presents the phase portraits for τ=1.335714, 1.435714, 1.485714, 1.515714, and 1.635714. The corresponding initial conditions are (*I*(0),*R*(0)) = (1.61,0.44), (1.61,0.22), (1.61,0.11), (1.61,0.165), and (2.00,0.35), respectively. For $\tau <\tau_{c}=1.535714$, the immune-free equilibrium *E*_0_ is unstable, whereas the interior equilibrium $E_{i}(\tau )$ is a nonlinear center surrounded by closed periodic orbits, indicating sustained oscillations of the immune cell populations. As τ approaches the critical value $\tau_{c}$ from below, the equilibrium moves toward the *R* = 0 axis since $R_{i}\to 0$ while $I_{i}=\lambda /\rho$ remains constant. At $\tau =\tau_{c}$, the interior equilibrium reduces to the immune-only boundary equilibrium $E_{b}=(\lambda /\rho ,0)$. For $\tau >\tau_{c}$, the interior equilibrium no longer exists, and all trajectories converge to the immune-free equilibrium *E*_0_, indicating elimination of the autoreactive immune cells.

**Fig 5:**
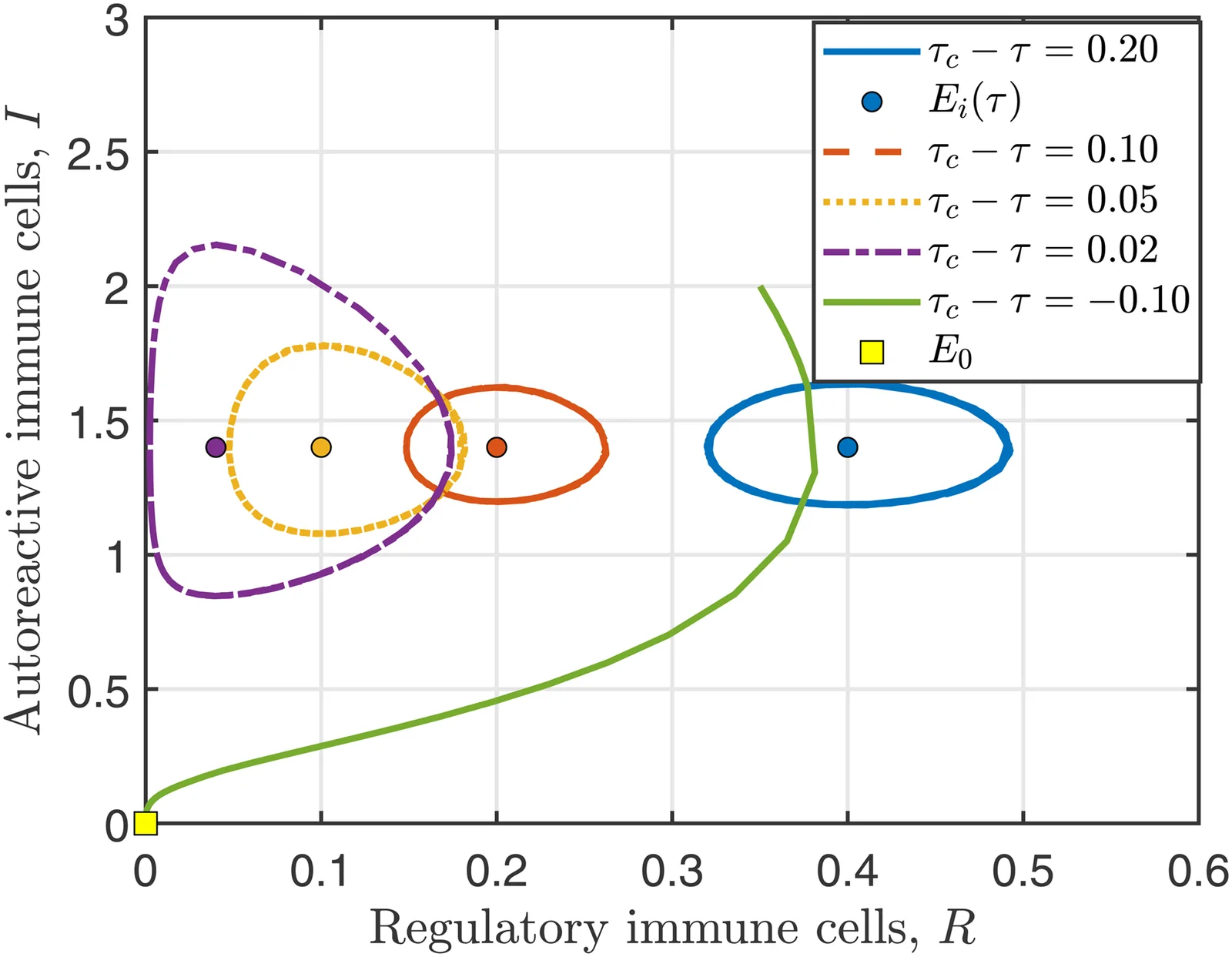
Phase portraits for different treatment levels τ. For $\tau <\tau_{c}$, the full-model trajectories exhibit recurrent oscillatory behavior consistent with the nonlinear-center and closed-orbit structure of the reduced immune subsystem. As $\tau \to \tau_{c}^{-}$, the interior equilibrium satisfies $R_{i}\to 0$ and approaches $E_{b}=(\lambda /\rho ,0)$, which lies on the critical equilibrium set ${ℰ}_{c}=\{(I,0):I\geq 0\}$. For $\tau >\tau_{c}$, the immune variables converge toward the immune-free equilibrium *E*_0_, indicating suppression of sustained autoreactive immune activity.

### 5.2 Sensitivity analysis of the autoimmune activation number at the immune-free state

To identify the parameters that most strongly influence the stability of the immune-free state, we investigate the sensitivity of the autoimmune activation number evaluated at the immune-free myelin level. Since the immune-free state corresponds to


$$\begin{array}{c} M_{0}=\frac{\alpha }{\delta }, \end{array}$$

the associated myelin damage level is


$$\begin{array}{c} D_{0}=M_{\max}-M_{0}=M_{\max}-\frac{\alpha }{\delta }. \end{array}$$

Consequently, the autoimmune activation number at the immune-free state is given by


$$\begin{array}{c} R_{0}(M_{0})=\frac{\eta (D_{0})}{\theta +\tau }=\frac{\eta_{\max}}{\theta +\tau }\frac{M_{\max}-\frac{\alpha }{\delta }}{\omega +M_{\max}-\frac{\alpha }{\delta }}. \end{array}$$

This threshold determines whether small perturbations in the autoreactive immune-cell population can grow near the immune-free state. Its sensitivity therefore provides insight into the parameters governing the onset of autoimmune activity.

#### Analytical sensitivity indices.

The normalized forward sensitivity index of $R_{0}(M_{0})$ with respect to a parameter *p* is defined by


$$\begin{array}{c} \Upsilon_{p}^{R_{0}}=\frac{p}{R_{0}(M_{0})}\frac{\partial R_{0}(M_{0})}{\partial p}. \end{array}$$

For positive parameter values and $R_{0}(M_{0})>0$, this is equivalently expressed as


$$\begin{array}{c} \Upsilon_{p}^{R_{0}}=\frac{\partial \ln R_{0}(M_{0})}{\partial \ln p}. \end{array}$$

This dimensionless quantity measures the relative response of the autoimmune activation number to a small relative change in the corresponding parameter.

Differentiating the expression for $R_{0}(M_{0})$ gives


$$\begin{array}{cc} \Upsilon_{\eta_{\max}}^{R_{0}} & =1,\;\Upsilon_{\theta }^{R_{0}}=-\frac{\theta }{\theta +\tau },\;\Upsilon_{\tau }^{R_{0}}=-\frac{\tau }{\theta +\tau },\\ \Upsilon_{M_{\max}}^{R_{0}} & =\frac{M_{\max}\delta^{2}\omega }{\left(\delta (M_{\max}+\omega )-\alpha )(M_{\max}\delta -\alpha \right)},\\ \Upsilon_{\alpha }^{R_{0}} & =-\frac{\alpha \delta \omega }{\left(\delta (M_{\max}+\omega )-\alpha )(M_{\max}\delta -\alpha \right)},\\ \Upsilon_{\delta }^{R_{0}} & =\frac{\alpha \delta \omega }{\left(\delta (M_{\max}+\omega )-\alpha )(M_{\max}\delta -\alpha \right)},\\ \Upsilon_{\omega }^{R_{0}} & =-\frac{\delta \omega }{\delta (M_{\max}+\omega )-\alpha }. \end{array}$$ (37)

Using the baseline parameter values listed in Table 2, with τ=0.5, the autoimmune activation number is $R_{0}(M_{0})=2.5287$. The corresponding normalized sensitivity indices are summarized in Table 3.

**Table 3:** Normalized sensitivity indices of the autoimmune activation number $R_{0}(M_{0})$ evaluated at the baseline parameter values.

Parameter *p*	$\Upsilon_{p}^{R_{0}}$
$\eta_{\max}$	1.0000
θ	−0.3333
τ	−0.6667
$M_{\max}$	0.0564
α	−0.0047
δ	0.0047
ω	−0.0517

The results in Table 3 indicate that $\eta_{\max}$, τ, and θ have the largest absolute sensitivity indices. In particular, the unit sensitivity of $\eta_{\max}$ shows that the autoimmune activation number changes proportionally with the maximum immune activation rate. The negative sensitivities associated with θ and τ indicate that increasing autoreactive immune-cell removal reduces $R_{0}(M_{0})$.

In contrast, $M_{\max}$, ω, α, and δ exhibit relatively small sensitivity magnitudes at the baseline parameter set. These findings identify immune activation and autoreactive immune-cell removal as the dominant mechanisms controlling the local disease threshold.

The analytical sensitivity indices characterize the response of $R_{0}(M_{0})$ to infinitesimal parameter perturbations at the baseline values. However, they do not describe the combined effects of simultaneous parameter variations over prescribed uncertainty ranges. To complement the analytical results, we perform a global sensitivity analysis.

#### Global sensitivity analysis using LHS–PRCC.

Global sensitivity analysis is conducted using Latin Hypercube Sampling (LHS) combined with Partial Rank Correlation Coefficients (PRCC). This approach assesses the relative influence of model parameters on the autoimmune activation number while allowing all selected parameters to vary simultaneously.

We consider the parameter vector


$$\begin{array}{c} p=(\eta_{\max},\theta ,\tau ,M_{\max},\alpha ,\delta ,\omega ), \end{array}$$

where each component is independently sampled within a prescribed interval around its baseline value $p_{i}^{*}$.

Because experimentally established uncertainty ranges are unavailable for several parameters, a variation of ±10% is adopted as an exploratory uncertainty interval:


$$\begin{array}{c} p_{i}\sim U(0.9p_{i}^{*},1.1p_{i}^{*}),\;i=1,...,7, \end{array}$$

where U denotes the uniform distribution.

A total of $N_{s}=2000$ parameter combinations are generated using LHS. For each sample, the autoimmune activation number $R_{0}(M_{0})$ is calculated using its analytical expression.

The PRCC between a parameter $p_{i}$ and $R_{0}(M_{0})$ is computed as


$$\begin{array}{c} \mathrm{PRCC}(p_{i},R_{0}(M_{0}))=\mathrm{Corr}\left(r_{p_{i}},r_{R_{0}}\right), \end{array}$$

where $r_{p_{i}}$ and $r_{R_{0}}$ denote the residuals obtained by regressing the rank-transformed values of $p_{i}$ and $R_{0}(M_{0})$, respectively, on the rank-transformed values of the remaining parameters.

PRCC values range between −1 and 1. Positive coefficients indicate a positive monotonic association with $R_{0}(M_{0})$, whereas negative coefficients indicate an inverse association. The absolute magnitude reflects the strength of the partial rank correlation.

Fig 6 illustrates the relative influence of the seven parameters on the autoimmune activation number.

**Fig 6:**
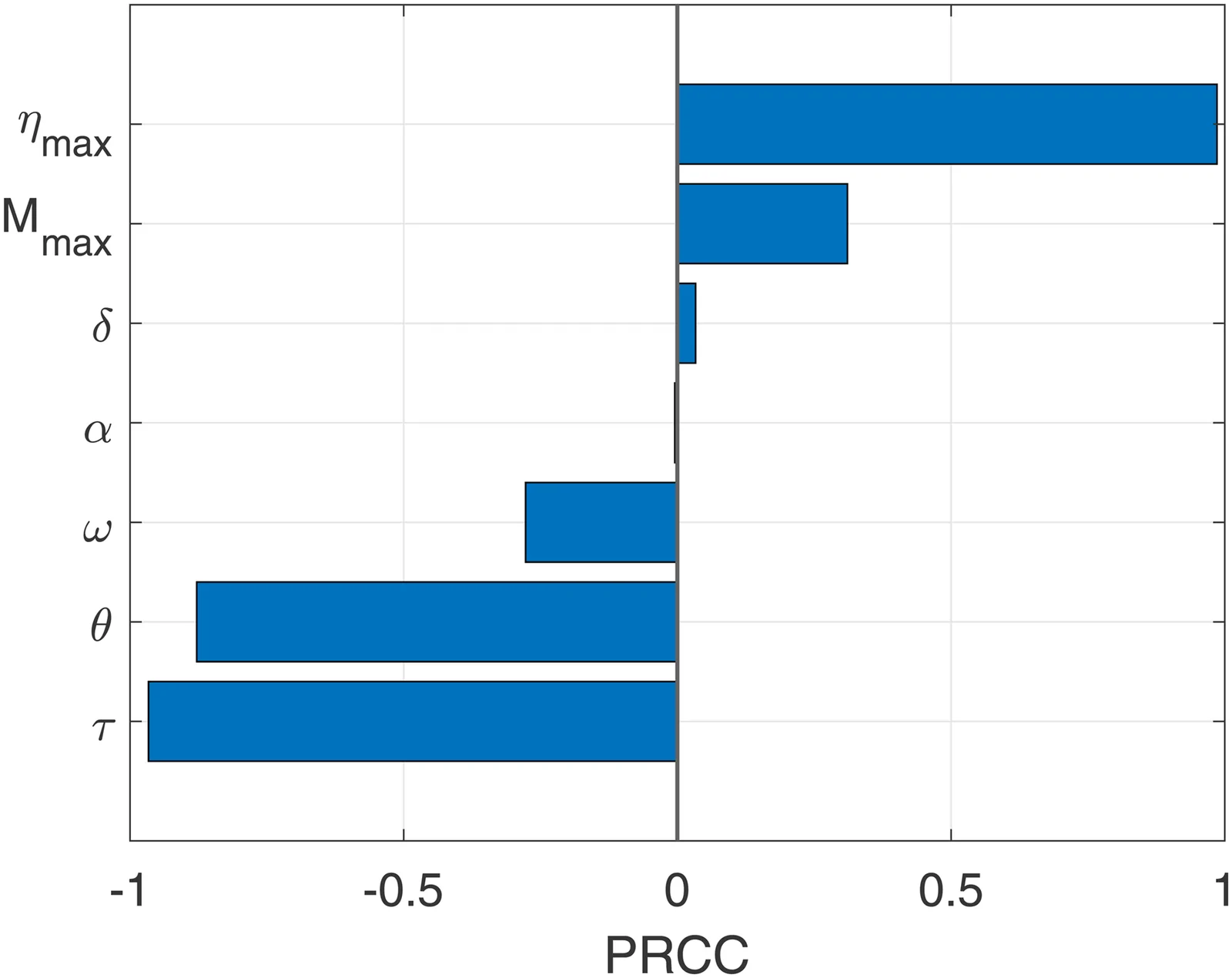
Global sensitivity analysis of the autoimmune activation number $R_{0}(M_{0})$ using Latin Hypercube Sampling and Partial Rank Correlation Coefficients. The seven model parameters are independently varied within ±10% of their baseline values.

The maximum immune activation rate $\eta_{\max}$ exhibits a strong positive correlation with $R_{0}(M_{0})$, whereas the removal parameters τ and θ exhibit strong negative correlations. These three parameters have the largest absolute PRCC values, indicating that they are the dominant contributors to variations in the autoimmune activation number over the investigated parameter ranges.

The parameters $M_{\max}$ and ω show weaker positive and negative associations, respectively. The PRCC values corresponding to α and δ are close to zero, suggesting limited influence within the considered uncertainty intervals.

The global sensitivity results are qualitatively consistent with the analytical sensitivity indices. In particular, both approaches identify immune activation and autoreactive immune-cell removal as the principal mechanisms affecting the autoimmune activation threshold.

This agreement supports the importance of targeting immune activation and autoreactive immune-cell clearance when developing strategies aimed at reducing the autoimmune activation number.

It should be emphasized that these conclusions are conditional on the chosen uncertainty intervals and the assumption of approximately monotonic parameter-output relationships. The analysis does not establish parameter influence outside the investigated ranges.

### 5.3 Optimal control solution

In this section, we compute the optimal treatment strategy for the proposed MS model by numerically solving the associated optimal control problem using a forward–backward sweep algorithm. Starting from an initial admissible control, the state system is solved forward in time while the adjoint system is solved backward in time. The control is updated at each iteration using the optimality condition (34) and projected onto the admissible control set. The iterative process continues until convergence is achieved, that is,


$$\begin{array}{c} \|u^{\left(k+1\right)}-u^{\left(k\right)}\|<\varepsilon_{1}, \end{array}$$

where $\varepsilon_{1}$ is a prescribed tolerance. The resulting optimal treatment is then used to compare disease dynamics with and without intervention. The comparison focuses on the evolution of autoreactive immune cells, the treatment intensity and its rate of adjustment, as well as the instantaneous and cumulative treatment costs. Numerical simulations show that the optimal therapy suppresses large inflammatory flares and maintains the immune activity at controlled levels while producing clinically realistic treatment profiles due to the inclusion of rate–smoothing penalties.

Fig 7 presents the optimal-control results, combining the immune-cell dynamics, cumulative objective value, treatment intensity, and treatment-adjustment rate into four panels.

**Fig 7:**
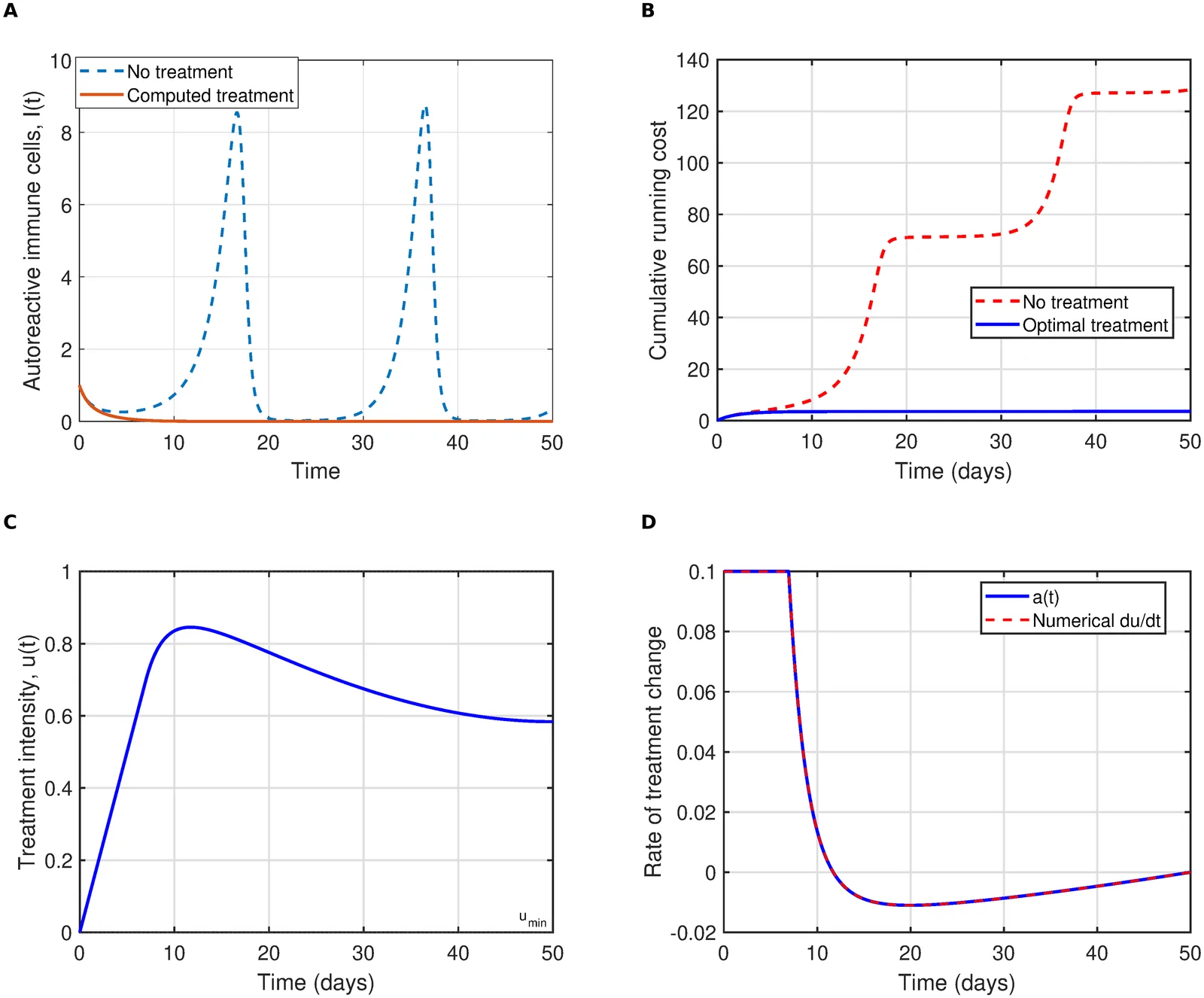
Optimal-control results for the multiple sclerosis model. (A) Autoreactive immune-cell dynamics with and without treatment. (B) Cumulative objective value for the untreated and optimally controlled cases. (C) Optimal treatment intensity *u*(*t*). (D) Comparison between the optimal treatment-adjustment rate *a*(*t*) and the numerical derivative *du*/*dt*, illustrating consistency with the treatment dynamics $\dot{u}(t)=a(t)$.

Panel A compares the evolution of the autoreactive immune-cell population *I*(*t*) under untreated and optimally controlled conditions. In the absence of treatment, the system exhibits pronounced oscillations associated with immune-regulatory feedback. Under optimal control, these oscillations are substantially reduced, and the autoreactive immune-cell population generally remains at lower levels. Temporary crossings between the controlled and uncontrolled trajectories may occur because the objective functional minimizes a weighted combination of cumulative immune burden and treatment costs rather than imposing pointwise minimization of *I*(*t*).

Panel B shows the cumulative objective value over the treatment horizon. Compared with the untreated case, the optimal strategy reduces the accumulated objective by limiting periods of elevated autoimmune activity while accounting for treatment intensity and adjustment costs. This comparison illustrates the effectiveness of the computed intervention within the proposed mathematical framework.

Panel C displays the optimal treatment intensity *u*(*t*). The computed strategy initially increases treatment intensity toward its admissible upper bound and subsequently maintains a relatively high level of intervention over much of the treatment horizon. This behavior reflects the balance between suppressing autoreactive immune activity and penalizing treatment exposure and rapid adjustments.

Panel D compares the optimal treatment-adjustment rate *a*(*t*) with the numerical derivative $\dot{u}(t)$ obtained from the treatment profile. Their close agreement provides a numerical consistency check for the implementation of the optimality system and the treatment dynamics $\dot{u}=a$.

Taken together, the four panels demonstrate that the computed optimal-control strategy substantially reduces recurrent autoimmune activity while accounting for treatment-related costs and adjustment constraints. These findings illustrate the potential of the proposed optimal-control framework to investigate treatment-dependent immune suppression within the mathematical model.

Overall, the effectiveness of the proposed treatment strategy is assessed by comparing the controlled and uncontrolled simulations. In the absence of treatment, recurrent autoimmune activity persists, leading to sustained inflammatory episodes and progressive myelin damage. Under the optimal treatment strategy, autoreactive immune activity is substantially suppressed, the cumulative objective value is reduced, and healthy myelin is better preserved. These numerical findings demonstrate the potential effectiveness of treatment-induced immune regulation within the proposed mathematical framework. The rate-smoothing mechanism further supports a gradual treatment profile by penalizing rapid adjustments in treatment intensity. Consequently, the optimal control framework provides a quantitative basis for evaluating treatment strategies and investigating their potential effects on autoimmune activity and disease progression.

#### 5.3.1 Effect of optimal treatment.

To quantify the impact of the proposed optimal control strategy, we compare the controlled solution with the untreated case over the simulation horizon. Following the approach in [8], we define the cumulative autoimmune burden by


$$\begin{array}{c} A^{t}=\int_{0}^{t_{f}}I(t)\;dt, \end{array}$$

where *I*(*t*) denotes the autoreactive immune-cell population. This quantity measures the total autoimmune activity accumulated over the treatment period.

The corresponding treatment efficiency is defined by


$$\begin{array}{c} F=\left(1-\frac{A^{t}}{A^{0}}\right)\times 100, \end{array}$$

where $A^{t}$ is the cumulative autoimmune burden under optimal treatment and *A*^0^ is the corresponding value in the absence of treatment. Thus, larger values of *F* indicate a greater reduction in cumulative autoimmune activity.

Table 4 summarizes the quantitative effect of the optimal treatment. In the absence of treatment, the cumulative autoimmune burden is


$$\begin{array}{c} A^{0}=64.22, \end{array}$$

whereas under optimal treatment it is reduced to


$$\begin{array}{c} A^{t}=1.73. \end{array}$$

**Table 4:** Effect of optimal treatment on cumulative autoimmune burden, treatment efficiency, and objective value.

Scenario	Cumulative burden *A*	Efficiency (%)	Objective *J*
No treatment	64.22	0	128.48
Optimal treatment	1.73	97.31	3.5676

This gives


$$\begin{array}{c} F=\left(1-\frac{1.73}{64.22}\right)\times 100\approx 97.31\%. \end{array}$$

Hence, the optimal treatment reduces the cumulative autoimmune burden by approximately 97.3% relative to the untreated case.

The objective value is also substantially reduced, from


$$\begin{array}{c} J^{0}=128.48 \end{array}$$

without treatment to


$$\begin{array}{c} J^{t}=3.5676 \end{array}$$

under optimal treatment. This reduction indicates that the controlled strategy achieves strong suppression of autoimmune activity while maintaining a relatively small treatment-adjustment cost.

The numerical trajectories in Fig 7 provide a complementary view of these results. In the untreated case, the autoreactive immune-cell population exhibits recurrent large-amplitude inflammatory peaks. Under optimal treatment, these oscillations are rapidly suppressed and the immune-cell population remains close to zero for most of the simulation interval.

The optimal treatment profile initially increases from zero, reaches its maximum level during the early phase of the intervention, and then gradually decreases as autoimmune activity is brought under control. The corresponding treatment-adjustment rate *a*(*t*) is initially positive, allowing treatment intensity to increase, and later becomes negative once treatment intensity begins to decrease. This behavior is consistent with the relation


$$\begin{array}{c} \dot{u}(t)=a(t). \end{array}$$

The close agreement between the optimal rate *a*(*t*) obtained from Pontryagin’s Maximum Principle and the numerical derivative *du*/*dt* provides an additional consistency check for the numerical solution.

Since autoreactive immune cells are the principal drivers of myelin destruction in the model, the substantial reduction in cumulative autoimmune activity is expected to reduce immune-mediated myelin damage. Overall, the results demonstrate that the proposed optimal-control strategy can strongly suppress autoimmune activity while avoiding unnecessarily large or abrupt changes in treatment intensity.

## 6 Conclusion

In this work, we developed and analyzed a mathematical model describing the interaction between autoreactive immune cells, regulatory immune responses, and myelin dynamics in multiple sclerosis. The model incorporates damage-dependent immune activation together with treatment-induced modulation, providing a biologically consistent and analytically tractable framework.

We established well-posedness of the system and derived a reduced immune subsystem by exploiting the separation of time scales between myelin and immune dynamics. This reduction enabled a rigorous analysis of equilibrium structure and stability properties. In particular, we identified the immune-free, boundary, and interior equilibria and characterized their existence and stability in terms of the system parameters.

A key contribution of this work is the identification of a treatment-dependent threshold structure governing the qualitative behavior of the reduced immune subsystem. While the autoimmune activation number $R_{0}$ determines the local stability of the immune-free equilibrium, the analysis also reveals distinct dynamical regimes on either side of the critical threshold $R_{0}=1$.

In particular, when $R_{0}>1$, the interior equilibrium exists as a nonlinear center and is surrounded by closed periodic orbits, corresponding to sustained autoimmune oscillations within the reduced immune subsystem. When $R_{0}<1$, the interior equilibrium no longer exists and the immune-free equilibrium is globally asymptotically stable in the biologically relevant domain of the reduced system.

At the critical threshold $R_{0}=1$, the reduced system possesses a continuum of boundary equilibria, with $E_{b}=(\lambda /\rho ,0)$ representing the limiting position of the interior equilibrium. These findings identify a critical treatment-dependent threshold separating persistent autoimmune activity from immune suppression and demonstrate how treatment alters the qualitative structure of the reduced immune dynamics.

Numerical simulations of the full model are qualitatively consistent with the analytical findings for the reduced immune subsystem and reveal distinct dynamical regimes. For $R_{0}>1$, the system exhibits recurrent inflammatory oscillations associated with progressive myelin damage. For sufficiently strong treatment, immune activity is suppressed and the system approaches the immune-free state.

Both analytical and global sensitivity analyses identify the maximum immune activation rate $\eta_{\max}$, the treatment-induced removal rate τ, and the natural removal rate θ as the most influential parameters affecting the autoimmune activation number. The agreement between normalized sensitivity indices and LHS–PRCC results highlights the importance of immune activation and autoreactive immune-cell removal in governing the disease threshold. These findings are conditional on the parameter ranges considered in the global sensitivity analysis.

An optimal control framework was formulated to identify treatment strategies that minimize disease burden while maintaining realistic therapeutic profiles. The numerical results indicate that the computed treatment strategies can reduce cumulative autoimmune activity while accounting for treatment intensity and adjustment costs in the objective functional.

Overall, the proposed framework integrates mechanistic modeling, threshold and stability analysis, local and global sensitivity analysis, and optimal control. The results provide insight into the role of immune activation, regulatory responses, and treatment in determining autoimmune persistence and suppression, while highlighting the importance of treatment-dependent changes in the qualitative structure of the reduced immune subsystem.

Parameter estimation, model calibration, and validation were not pursued in the present study because suitable clinical or experimental datasets containing all model variables are not currently available. The primary objective was to develop and analyze a mechanistic mathematical framework capable of capturing qualitative features of immune dynamics in multiple sclerosis. Consequently, the numerical findings should be interpreted as mechanistic predictions rather than patient-specific clinical outcomes.

Moreover, the present study is based on a deterministic ordinary differential equation framework and therefore does not explicitly account for random fluctuations associated with immune heterogeneity, environmental variability, or uncertainty in treatment response.

In addition, the neuronal equation represents cumulative neuronal loss and does not include neuronal repair or feedback from neuronal damage to the immune dynamics. Incorporating neuronal recovery and bidirectional neuroimmune feedback would therefore be a relevant extension of the present framework.

Future work will focus on parameter estimation and validation using suitable clinical datasets, together with extensions incorporating additional immune pathways, patient heterogeneity, and time delays associated with immune activation, regulatory responses, demyelination, remyelination, and neuronal damage. Stochastic formulations may also be investigated to examine noise-induced transitions, relapse–remission patterns, and uncertainty in treatment outcomes. Such developments may improve the predictive capability and biological relevance of the proposed framework and reveal additional dynamical behaviors, including delay-induced oscillations and bifurcations. These directions are motivated by recent advances in stochastic disease modeling, delay differential equations, Hopf bifurcation analysis, and optimal control of demyelinating diseases [9–12].
